# Distributed and retinotopically asymmetric processing of coherent motion in mouse visual cortex

**DOI:** 10.1038/s41467-020-17283-5

**Published:** 2020-07-16

**Authors:** Kevin K. Sit, Michael J. Goard

**Affiliations:** 1 Department of Psychological and Brain Sciences, University of California, Santa Barbara, Santa Barbara, CA 93106 USA; 2 Department of Molecular, Cellular, and Developmental Biology, University of California, Santa Barbara, Santa Barbara, CA 93106 USA; 3 Neuroscience Research Institute, University of California, Santa Barbara, Santa Barbara, CA 93106 USA

**Keywords:** Neuroscience, Sensory processing, Visual system, Extrastriate cortex, Motion detection

## Abstract

Perception of visual motion is important for a range of ethological behaviors in mammals. In primates, specific visual cortical regions are specialized for processing of coherent visual motion. However, whether mouse visual cortex has a similar organization remains unclear, despite powerful genetic tools available for measuring population neural activity. Here, we use widefield and 2-photon calcium imaging of transgenic mice to measure mesoscale and cellular responses to coherent motion. Imaging of primary visual cortex (V1) and higher visual areas (HVAs) during presentation of natural movies and random dot kinematograms (RDKs) reveals varied responsiveness to coherent motion, with stronger responses in dorsal stream areas compared to ventral stream areas. Moreover, there is considerable anisotropy within visual areas, such that neurons representing the lower visual field are more responsive to coherent motion. These results indicate that processing of visual motion in mouse cortex is distributed heterogeneously both across and within visual areas.

## Abstract (web summary)

Processing of coherent motion has been extensively studied in the primate visual system, but has not been well characterized in mice. Here, the authors use widefield calcium imaging to reveal that coherent motion responses are organized anisotropically both across and within visual areas in mice.

## Introduction

Perception of visual motion is critical for animal survival, underlying behaviors such as visually guided navigation, pursuit of prey, and avoidance of threats. Although neurons selective for visual motion arise early in the visual system, extensive research in primates has shown that perception of coherent global motion independent of local motion relies on processing in specialized regions of visual cortex^1,2^. Although mouse visual cortical neurons are known to be well-tuned for coherent visual motion^3,4^, and vision plays an important role in navigation^5^, mesoscale cortical processing of coherent motion is poorly understood in the mouse. Recently developed techniques for measuring neural activity in genetically identified neurons makes the mouse an attractive model system for investigating cortical processing of coherent motion at previously inaccessible spatial scales^6,7^. Although visually driven neurons along the retinogeniculocortical pathway of mice and primates exhibit differences in response properties and connectivity^8^, there are parallels in the overarching meso- and macroscale organization of the visual areas^6^. Researchers are just beginning to understand how coherent motion is encoded in the mouse visual system, and the degree to which circuits underlying motion processing are homologous between mice and primates.

In primates, the middle temporal area (MT) and the downstream medial superior temporal area (MST) have been identified as specialized regions for processing of coherent motion^9^, and are key regions in the dorsal stream of visual processing^10,11^. Area MT contains a high proportion of direction-selective neurons^12–14^ and preferentially receives direction-selective inputs from the primary visual cortex (V1)^15^. Pharmacological lesioning of MT causes deficits in coherent motion perception^16^, and microstimulation of MT can influence perception of motion in studies using random dot kinematograms (RDKs)^17^. Individual neurons in MT and MST exhibit strong direction-selective responses to RDKs and many are selective for the overall motion of plaid stimuli (pattern direction-selective) rather than to the individual component gratings (component direction-selective)^9^. In contrast, neurons in the primate V1 are mostly nonselective for the direction of coherent motion in RDKs^18^ and exclusively exhibit component direction-selective responses to plaids^9^. These findings have led to models in which MT response properties derive from weighted summation and normalization of direction-selective V1 inputs^19,20^.

The basic organization of mouse visual cortex is similar to the primate visual system, with the majority of cortical input arriving via the retinogeniculocortical pathway (along with indirect input from the superior colliculus via the lateral posterior nucleus of the thalamus^21^), and a network of hierarchically organized visual cortical regions with independent retinotopic maps^22–24^. However, the mouse visual system has several functional properties that are distinct from that of primates. For example, several types of mouse retinal ganglion cells (RGCs) exhibit direction selectivity^25–27^, while direction-selective RGCs have not been found in primate retina^28^. Direction-selective RGCs have an asymmetric retinotopic distribution^29,30^, give rise to direction-selective inputs to visual cortex^31^, and influence direction selectivity in visual cortex^32^. In addition, strong orientation tuning and direction selectivity are already present in the lateral geniculate nucleus^33–35^, in contrast to the weakly tuned LGN neurons found in primates^36^. Finally, in contrast to primate V1^9,18^, mouse V1 contains a significant fraction of neurons that exhibit tuned responses to global coherent motion found in RDKs^3^ and plaid pattern motion^37,38^ (though see ref. ^39^).

In recent years, mapping procedures using intrinsic signal imaging^23,24,40^ and wide-field calcium imaging^41,42^ have allowed researchers to define and functionally characterize higher visual areas (HVAs) in intact mice, but the functional role of the HVAs and any homology to primate visual structures remains an area of active investigation. Anatomical and functional studies have found that HVAs are broadly connected into two subnetworks with projection patterns similar to primate ventral and dorsal streams^40,43–45^. Specifically, the lateral medial (LM) and lateral intermediate (LI) areas preferentially project to temporal and lateral entorhinal cortices (putative ventral stream) while the anterolateral (AL), posterior medial (PM), rostrolateral (RL), and anteromedial (AM) areas preferentially project to parietal, motor, and medial entorhinal cortices (putative dorsal stream). Consistent with this classification, measurements of single neuron activity indicated greater direction selectivity in regions AL, RL, and AM^23^ (though see ref. ^24^), a hallmark of dorsal stream regions in primate^12–14^.

Here, we use wide-field and two-photon calcium imaging to map areal and cellular responses to coherent motion in mouse visual cortices using both natural movies and RDKs. We find that HVAs exhibit heterogeneous responses to coherent motion as in primates, with stronger activation in response to coherent motion in regions AL, PM, and AM compared with V1, LM, and LI. However, responses to coherent motion are much more distributed than in primate, with neurons in all measured regions (including V1) exhibiting some degree of coherent motion responsiveness. Furthermore, coherent motion responses are distributed asymmetrically across visual elevation, both within and across all visual regions, with neurons representing the lower visual field exhibiting much stronger coherent motion responses. Taken together, these results show that the mouse visual cortex is optimized for distributed processing of lower field motion, potentially enhancing processing of optical flow signals during movement.

## Results

### Mouse visual cortex has heterogeneous responses to motion

**Fig. 1:**
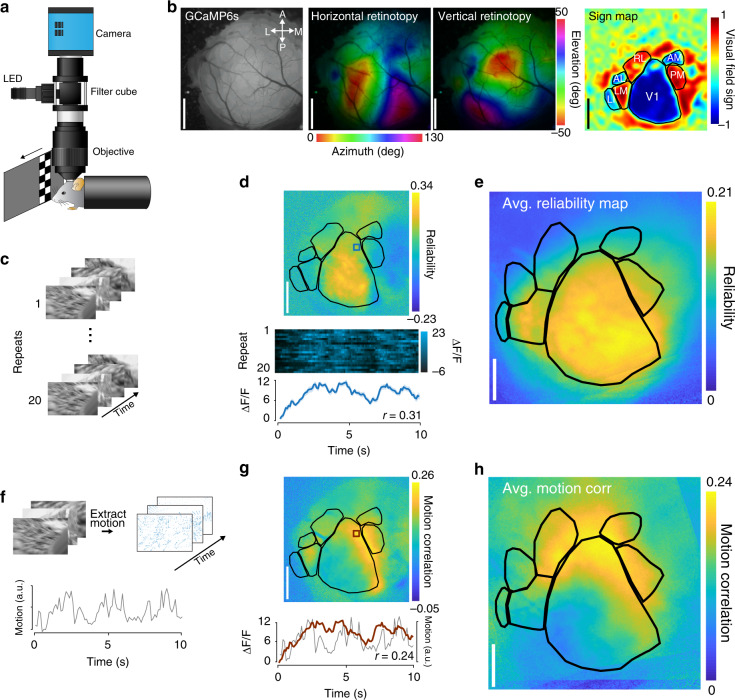
Mesoscale calcium responses to motion energy in natural visual stimuli. **a** Schematic of the custom epifluorescent wide-field microscope for in vivo GCaMP6s imaging. The screen depicts a retinotopic mapping stimulus, with a drifting bar moving across the visual field (azimuth mapping). **b** Areal maps from one session of a single example mouse. Left: surface raw fluorescence image of a 4 mm cortical window of example Emx1-GCaMP6s mouse. Middle: horizontal and vertical retinotopic maps showing preferred location of each pixel for azimuth (left) and elevation (right); color bars indicate degree offset from center of visual field). Right: sign map (red, positive; blue, negative), and resulting segmentation of visual cortex into V1 and HVAs. Scale bars = 1 mm. **c** Schematic of the natural movie stimulus. Scenes were repeated 20 times to measure reliable neural responses. **d** Top: map from a single experiment showing reliability across posterior cortex; visual area segmentation as in (**b**). Bottom: multi-trial response (20 repeats) and mean trace (±s.e.m. shaded) of a single pixel (blue square) to repeated presentation of the natural movie. Reliability is defined as the across-trial Pearson correlation coefficient (*r* = 0.31, see Methods). **e** Mean reliability map across all imaged mice (*n* = 19 sessions over 7 mice). Individual maps are transformed onto a common coordinate system for comparison across mice. **f** Extraction of the motion energy in the stimulus. Pixel-wise motion vectors were extracted from each frame of the movie, and the sum of these vectors is used as a measure of the net motion energy of each frame. **g** Top: map from a single experiment showing motion response across posterior cortex; visual area segmentation as in (**b**). Bottom: Neural response of a single pixel (red) overlaid on the motion trace (gray); pixel-wise motion energy correlation is calculated as the Pearson correlation between these two signals (*r* = 0.24). **h** Mean motion energy correlation map across all imaged mice (*n* = 19 sessions over 7 mice); alignment procedure same as (**e**). Area abbreviations: primary visual (V1), lateral medial (LM), anterolateral (AL), posterior medial (PM), laterointermediate (LI), rostrolateral (RL), and anteromedial (AM).

In order to measure neural responses to coherent motion in an unbiased manner across the visual cortex, we used a custom wide-field microscope (Fig. 1a) to measure calcium responses through a 4-mm diameter window located over the left visual cortex of awake, head-restrained mice expressing the calcium indicator GCaMP6s in excitatory neurons (Emx1-Cre::Rosa-tTA::TITL-GCaMP6s^46,47^; Supplemental Video S1). We displayed visual stimuli on a screen that was placed on the optical axis of the right eye, such that the center of gaze was centered on the display monitor (Supplementary Fig. 1, see Methods). Using established mapping procedures for defining HVAs^23,40^ that were adapted for calcium imaging^41,42^, we determined the areal boundaries of primary visual cortex (V1) and six consistently identified HVAs: LM, AL, PM, LI, RL, and AM (Fig. 1b), ordered by their approximate position in the visual hierarchy^44^. As in some previous studies, we were unable to consistently locate area A independent of AM^40^, so this area was excluded from our analyses. This procedure was performed separately for each mouse to obtain a precise map of each mouse’s visual cortical areas (Supplementary Fig. 2). To determine which areas responded to complex visual input, we displayed repeated presentations of sets of natural movies recorded from a head-mounted camera^48^ (Fig. 1c) on a large monitor subtending 130° azimuth (0–130° nasal to temporal) and 100° elevation (−50° to +50° lower to upper) of the contralateral visual field. Calcium responses from individual pixels in visual cortex exhibited reliable responses to repeated presentations of the natural movies (Fig. 1d). To reduce inter-mouse variability and hemodynamic artifacts from blood vessels, we aligned HVA area boundaries and averaged reliability across multiple mice (Fig. 1e; *n* = 19 sessions across 7 mice), revealing that primary visual cortex exhibited uniformly reliable responses across the region. Response reliability was slightly weaker in secondary visual regions LM and PM, and weaker still in higher areas of the visual hierarchy such as AL, RL, and AM (Supplementary Fig. 3).

We next investigated whether the responses to the motion energy embedded within the natural movies were similarly uniform across areas, or if the mouse exhibits heterogeneity across HVAs as has been observed in primate MT/MST. To this end, we first calculated the total motion energy for each frame of the natural movie by first calculating pixel-by-pixel motion vectors, then taking the vector sum across each frame (Fig. 1f; see Methods). We next measured the Pearson correlation between the deconvolved neural response for each pixel and the motion energy of the scene (Fig. 1g, bottom; see Methods). In both individual sessions (Fig. 1g, top) and aligned session averages (Fig. 1h; *n* = 19 sessions across 7 mice), we found that particular cortical locations were strongly driven by motion embedded in the scene. The motion correlation map (Fig. 1h) was not well correlated to the map of reliability to all visual features (Fig. 1e), as evident by the low pixel-wise correlation between motion response and reliability (Supplementary Fig. 4*; r* = 0.088 ± 0.12, *t*_17_ = 0.08, *p* = 0.47, mean ± s.e.m., single-sample *t* test).

**Fig. 2:**
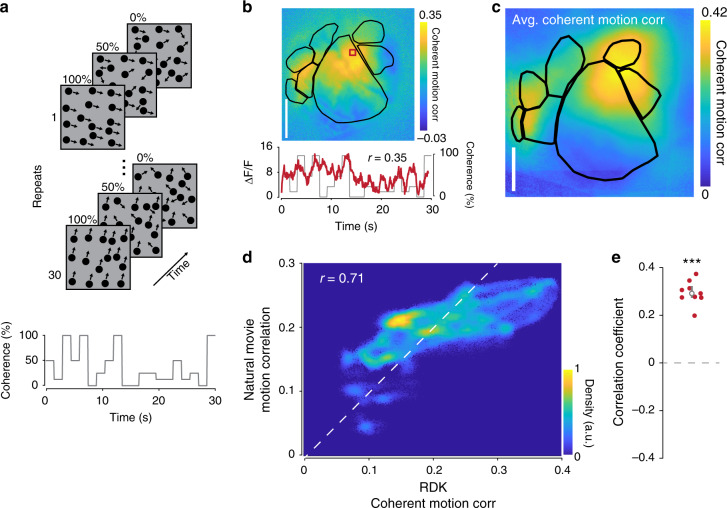
Mesoscale calcium responses to coherent motion in RDKs. **a** Schematic of the RDK stimulus. A single repeat of the stimulus contains dots drifting at varying coherence values across the trial duration (bottom). Over multiple repeats, the coherence values remain constant while the dot positions and directions are randomized every trial. **b** Top: map from a single experiment showing coherent motion correlation to RDKs across posterior cortex. Bottom: single pixel response (red) over the course of a single presentation of the stimulus overlaid on the coherence value trace; pixel-wise coherent motion correlation is calculated as the Pearson correlation between these two signals (*r* = 0.35). **c** Mean coherent motion correlation map across all imaged mice (*n* = 10 sessions over 10 mice). Individual maps are transformed onto a common coordinate system for comparison across mice. **d** Pixel-wise density plot of RDK vs. natural movie motion correlation across all mice to show similarity of motion correlation across visual stimuli (*r* = 0.71). Density plots are similar to scatter plots but provide additional information about the density (but not strength) at each coordinate, with warmer colors indicating a higher density of observation pairs (see Methods). **e** Natural movie versus RDK motion correlation for each mouse (*r* = 0.30 ± 0.02, *n* = 10, *p* = 1.0 × 10^−7^; two-tailed single-sample *t* test).

One possibility for the anisotropic distribution of motion responses in visual cortex is that motion energy is not uniformly distributed in the natural movies. Indeed, individual natural movies exhibit non-uniform distributions of motion energy (Supplementary Fig. 5), though the average motion energy across all natural movies is roughly uniform (Supplementary Fig. 5). To further ensure that the distribution of motion energy is unbiased across the visual field, and to remove the influence of other features driving neural responses, we used full screen random dot kinematograms (RDKs) to measure responses to coherent motion across the visual field (Fig. 2a; see Methods).

The calcium response within a single pixel represents the summed activity across many neurons, so we could not measure tuning to preferred motion direction as is typically done in single neuron recordings. Instead, we took advantage of the fact that RDKs with higher coherence values will strongly drive neurons responsive to coherent motion and result in higher magnitude calcium responses regardless of motion direction. We used a small dot size (2° of visual space) and randomized dot position and drift direction on each trial, so that the only consistent visual feature across trials was the RDK motion coherence. We then defined the coherent motion correlation for each pixel as the Pearson correlation coefficient between the pixel response (Δ*F*/*F*) and the RDK motion coherence (see Methods). We found that individual pixels in V1 and other HVAs exhibited a high degree of correlation with RDK coherence (Fig. 2b). Aligning and averaging motion correlation maps revealed a anisotropic distribution of coherent motion correlated pixels within and across regions (Fig. 2c). Despite the difference in stimulus, the motion correlation map measured with RDKs (Fig. 2c) was well correlated to the motion correlation map measured with natural movies (Fig. 1g), as indicated by the Pearson correlation of aligned pixels (Fig. 2d, e*; r* = 0.71; *t*_9_ = 14.0, *p* = 1.0 × 10^−7^, single-sample *t* test), although certain regions (e.g., RL) showed differences between stimuli (see Discussion).

### HVAs exhibit differential responses to coherent visual motion

In primates, particular higher visual areas (MT/V5 and MST) are specialized for processing of coherent motion. To determine if particular mouse HVAs exhibit similarly dedicated motion processing, we examined coherent motion correlations in V1, each consistently defined HVA (LM, PM, AL, LI, RL, and AM), and somatosensory region S1 (as a negative control).

**Fig. 3:**
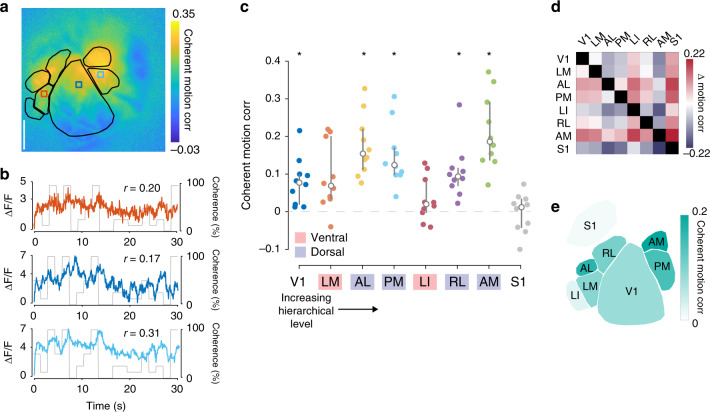
HVAs exhibit anisotropic coherent motion correlations to RDKs. **a** Map from a single experiment highlighting anisotropic coherent motion correlations across HVAs. **b** Single pixel responses taken from area LM (orange; top), V1 (dark blue; middle), and PM (light blue; bottom) overlaid on the coherence trace. Correlation between pixel response and coherent motion trace indicated for each pixel. **c** Across all mice (*n* = 10 sessions over 10 mice), each tested dorsal stream visual area is significantly motion responsive, but neither ventral stream areas nor a control area outside of visual cortex (region S1) are motion responsive (*Bonferroni adjusted *p* < 0.006 (*p* = 0.05, 8 tests); two-tailed single-sample *t* test). Overlay indicates median ± quartiles. **d** Relative difference matrix, with each element equal to the coherent motion correlation in the column region subtracted from the coherent motion correlation in the row region across mice (red, higher; blue, lower). **e** Simplified schematic of mean coherent motion correlations in each cortical area across mice.

For each session, we defined visual areas using retinotopic mapping, and then measured coherent motion correlations for each pixel as the Pearson correlation between the deconvolved Δ*F*/*F* signal and the RDK motion coherence (Fig. 3a, b). We then averaged all the pixels within each defined region of interest to generate a measure of coherent motion correlation by area. Using a Bonferroni-corrected *p*-value of 0.006 (*p* = 0.05, 8 tests), our statistical comparisons revealed that all visual regions except LM and LI exhibited coherent motion correlation values significantly above zero (Fig. 3c, V1: 0.08 ± 0.02, *t*_9_ = 3.6, *p* = 3.0 × 10^−3^; LM: 0.09 ± 0.03, *t*_9_ = 2.8, *p* = 9.7 × 10^−3^; AL: 0.17 ± 0.03, *t*_9_ = 5.9, *p* = 1.1 × 10^−4^; PM: 0.15 ± 0.03, *t*_9_ = 5.1, *p* = 3.0 × 10^−4^; LI: 0.03 ± 0.02, *t*_9_ = 1.3, *p* = 0.11; RL: 0.11 ± 0.02, *t*_9_ = 4.2, *p* = 1.1 × 10^−3^; AM: 0.21 ± 0.03, *t*_9_ = 6.0, *p* = 1.0 × 10^−4^; Bonferroni-corrected threshold *p* < 0.006, mean ± s.e.m., single-sample *t* test). As expected, area S1 was not motion responsive (0.003 ± 0.01, *t*_9_ = 0.2, *p* = 0.59; mean ± s.e.m., single-sample *t* test). Moreover, some HVAs had significantly higher coherent motion correlation than other visual regions (Fig. 3d). Specifically, area AM had significantly higher motion response values than all other regions (*t*_68_ = 3.3, *p* = 6.9 × 10^−4^, Hedges’ *g* = 1.2, unpaired two-sample *t* test), followed by AL, PM, RL, V1, and LM, while area LI had the lowest coherent motion correlation (*t*_68_ = −3.2, *p* = 9.9 × 10^−4^, Hedges’ *g* = 1.2, unpaired two-sample *t* test, schematized in Fig. 3e). These results indicate that particular HVAs in mice indeed exhibit enhanced responses to coherent motion relative to primary visual cortex. Given the differences in coherent motion correlations in HVAs, these results are broadly consistent with past anatomical and functional work suggesting that areas LM and LI constitute a homolog of the ventral stream, while areas AL, PM, RL, and AM constitute a homolog of the dorsal stream^43–45^. Indeed, each imaged dorsal stream area is significantly responsive to coherent motion, whereas ventral stream areas are more varied. In addition, dorsal areas show an elevated coherent motion correlation as compared with ventral stream areas as a whole (dorsal: 0.16 ± 0.04, ventral: 0.06 ± 0.06, *t*_68_ = 3.9*, p* = 1.1 × 10^−4^, Hedges’ *g* = 1.1, mean ± s.e.m., unpaired two-sample *t* test).

### Coherent motion correlations exhibit retinotopic asymmetry

**Fig. 4:**
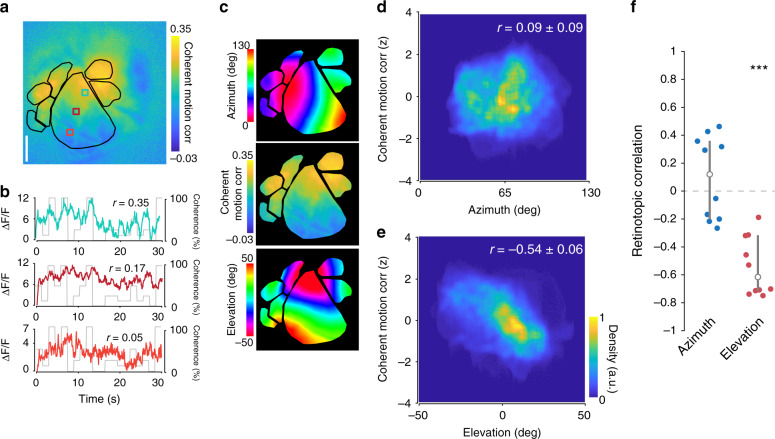
Retinotopic asymmetry of coherent motion correlations across the visual cortex. **a** Map from single experiment showing gradient of coherent motion correlation along the vertical retinotopic axis of V1. **b** Single pixel responses from anterior (blue, *r* = 0.35), middle (red, *r* = 0.17), and posterior (orange, *r* = 0.05) pixels in V1, showing different amounts of coherent motion correlation. **c** Visual cortex isolated maps from mouse in (**a**) of azimuth (top) and elevation (bottom), as well as the coherent motion correlation (middle) for comparison. **d** Density scatter plot across all mice (*n* = 10 sessions over 10 mice), showing no significant correlation to azimuth (*r* = 0.09 ± 0.09). Coherent motion correlation is *z*-scored to account for differences between experiments. **e** Same as (**d**), but for elevation, showing a significant negative correlation between elevation preference and coherent motion correlation across visual cortex (*r* = −0.54 ± 0.06, *p* = 1.9 × 10^−5^, two-tailed single-sample *t* test). **f** Comparison of retinotopic correlation between azimuth and elevation. There is a significant anticorrelation of coherent motion correlation and elevation across mice, but none with azimuth (*p* = 1.9 × 10^−5^, two-tailed single-sample *t* test. Error bars are median ± quartiles (***p < 0.001).

In addition to differences in coherent motion correlations between visual areas, there was also anisotropy within individual visual areas. To quantify this, we first considered area V1, where pixels located in anterior V1 were more strongly driven by coherent motion than pixels in posterior V1 (Fig. 4a, b). The anisotropy of coherent motion correlations in V1 could be explained in two ways: an anatomy-based organization (along anatomical axes) or a functional-based organization (along retinotopic axes). To test these possibilities, we isolated the entire visual cortex and calculated the *z*-scored coherent motion correlation for each pixel as a function of azimuth and elevation, as mapped with drifting bar stimuli (Fig. 4c). Although there was little correlation across mice (*n* = 10 sessions over 10 mice) between the *z*-scored motion correlation and the azimuth, there was a strong negative correlation between the coherent motion correlation and the elevation, indicating stronger coherent motion processing in the lower visual field (Fig. 4d–f; azimuth *r* = 0.09 ± 0.09, *t*_9_ = 0.4, *p* = 0.34; elevation *r* = −0.54 ± 0.06, *t*_9_ = −7.5, *p* = 1.91 × 10^−5^; single-sample *t* test).

To confirm that the anisotropy of coherent motion correlations in visual cortex is robust across measures, we also calculated coherent motion response curves for each pixel, fit a slope to each response curve, and plotted a map of the slopes (Supplementary Fig. 6A, B). This analysis resulted in qualitatively similar distribution of areal coherent motion responses (Supplementary Fig. 6C, D) and a negative correlation between coherent motion response and elevation (Supplementary Fig. 6E, F).

Because the stimulus monitor is flat, the edges of the display are farther from the mouse’s eye than the center of the screen. Given that the dots in the RDK are of uniform size, the perceived size of the dots at the edges of the screen could be below the visual acuity of the mouse visual system. To confirm that our results are not due to this effect, we performed two additional control experiments.

First, we examined the retinotopy of RDK-driven activity independent of coherent motion (Supplementary Fig. 7). If the size of the dots near the edge of the screen were below the threshold of visual acuity, we would expect a radially decreasing magnitude from the center of V1 to the periphery. However, we do not observe this pattern (Supplementary Fig. 7A–C). All visual areas but RL show increased activity due to visual stimulus onset and area S1 shows a mild depression (Supplementary Fig. 7D, V1: 10.43 ± 0.75, *t*_9_ = 14.4, *p* = 8.1 × 10^−8^; LM: 2.78 ± 0.30, *t*_9_ = 9.5, *p* = 2.7 × 10^−6^; AL: 2.03 ± 0.43, *t*_9_ = 4.7, *p* = 5.4 × 10^−4^; PM: 4.59 ± 0.49, *t*_9_ = 5.3, *p* = 2.4 × 10^−4^; LI: 1.48 ± 0.28, *t*_9_ = 5.5, *p* = 2.0 × 10^−4^; RL: 0.30 ± 0.23, *t*_9_ = 1.0, *p* = 0.18; AM: 1.29 ± 0.21, *t*_9_ = 6.1, *p* = 8.6 × 10^−5^; S1: −0.57 ± 0.16, *t*_9_ = −3.5, *p* = 3.3 × 10^−3^; mean ± s.e.m., single-sample *t* test). We also observed a weak positive correlation between visually driven activity and both azimuth and elevation (Supplementary Fig. 7E, F). However, this pattern is incongruent with the pattern described for coherent motion correlation (Fig. 4a).

To further confirm that the retinotopic anisotropy was not due to variable screen distance, in a subset of mice we applied spherical correction to the RDKs (Supplementary Fig. 8A), resulting in uniform perceived dot size across the stimulus display. The resulting coherent motion correlation maps are nearly identical to those using the uncorrected RDKs (Supplementary Fig. 8B, C). Furthermore, the correlation between coherent motion correlation and elevation remains strongly negative, while remaining uncorrelated for azimuth (Supplementary Fig. 8D, E).

**Fig. 5:**
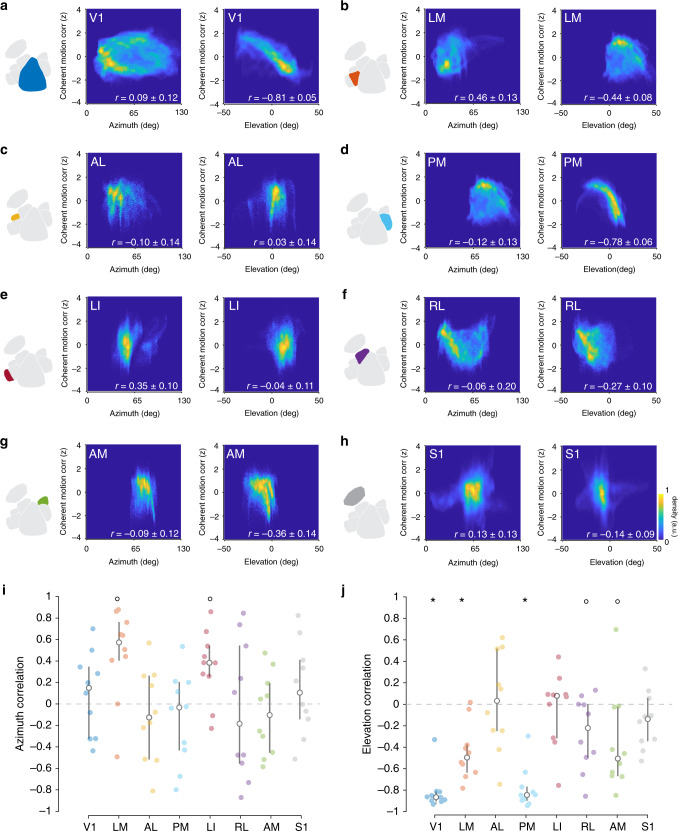
Retinotopic dependence of coherent motion correlation in each visual area. **a** Left: combined density scatter plot across all mice (*n* = 10 sessions over 10 mice) comparing *z*-scored coherent motion correlation to azimuth (Pearson’s correlation coefficient *r* = 0.09, *p* = 0.44) for area V1. Right: same as left, but for elevation (*r* = −0.81, *p* < 0.001). **b**–**h** Same as (**a**) for each HVA (LM, AL, PM, LI, RL, AM), and somatosensory cortex (S1). **i** Mean Pearson’s correlation coefficient between azimuth and coherent motion correlation for each area; only area LM and LI have a significant correlation (V1: *p* = 0.44; LM: *p* = 7.0 × 10^−3^; AL *p* = 0.46; PM: *p* *=* 0.38; LI: *p* *=* 8.0 × 10^−3^; RL: *p* *=* 0.76; AM: *p* *=* 0.46; S1: *p* *=* 0.33; two-tailed single-sample *t* test). **j** Same as (**i**), but for elevation. All visual areas except AL (*r* = 0.03, *p* = 0.85) and LI (*r* = −0.04, *p* = 0.74) show significant negative correlation between elevation and coherent motion correlation Control area S1 does not exhibit a correlation for either azimuth or elevation (V1: *p* = 1.4 × 10^−6^; LM: *p* = 4.3 × 10^−3^; AL: *p* = 0.85; PM: *p* = 4.5 × 10^−6^; LI: *p* *=* 0.73; RL: *p* = 0.035; AM: *p* = 0.036; S1: *p* = 0.1; two-tailed single-sample *t* test). °*p* < 0.05; *Bonferroni adjusted *p* < 0.006, *p* = 0.05, 8 tests). Error bars are median ± quartiles.

We next investigated whether the retinotopic asymmetry was also present in other HVAs. For each area, we calculated the correlation between the *z*-scored coherent motion correlation and azimuth (Fig. 5a–h, left plots) and elevation (Fig. 5a–h, right plots). No visual areas exhibited significant correlation between the preferred azimuth and the coherent motion correlation, although areas LM and LI trended toward a significant positive correlation (Fig. 5i; V1: 0.09 ± 0.12*, t*_9_ = 0.2, *p* = 0.44; LM: 0.46 ± 0.13, *t*_9_ = 3.0, *p* = 7.0 × 10^−3^; AL: −0.10 ± 0.14, *t*_9_ = −0.1, *p* = 0.46; PM: −0.12 ± 0.13, *t*_9_ = −0.3, *p* = 0.38; LI: 0.35 ± 0.10, *t*_9_ = 3.0, *p* = 8.0 × 10^−3^; RL: −0.06 ± 0.20, *t*_9_ = −0.7, *p* = 0.76; AM: −0.09 ± 0.12, *t*_9_ = −0.1, *p* = 0.46; S1: 0.13 ± 0.13, *t*_9_ = 0.5, *p* = 0.33; Bonferroni-corrected threshold *p* < 0.006, mean ± s.e.m., single-sample *t* test). Conversely, several regions (areas V1, PM, and LM) exhibited a significant negative correlation between the preferred elevation and the coherent motion correlation. In addition, areas AM and RL were trending toward significance, but areas AL, LI, and S1 did not show significant retinotopic anisotropy in coherent motion correlation (Fig. 5j; V1: −0.81 ± 0.05, *t*_9_ = 5.3, −10.3, *p* = 1.4 × 10^−6^; LM: −0.44 ± 0.08, *t*_9_ = −3.3, *p* = 4.3 × 10^−3^; AL: 0.03 ± 0.14*, t*_9_ = −1.1, *p* = 0.85; PM: −0.78 ± 0.06, *t*_9_ = −8.9, *p* = 4.5 × 10^−6^; LI: −0.04 ± 0.11, *t*_9_ = −0.6, *p* = 0.73; RL: −0.27 ± 0.10, *t*_9_ = −2.1, *p* = 0.035; AM: −0.36 ± 0.14, *t*_9_ = −2.0, *p* = 0.036; S1: −0.14 ± 0.09, *t*_9_ = −1.3, *p* = 0.11; Bonferroni-corrected threshold *p* < 0.006, mean ± s.e.m., single-sample *t* test). This pattern of coherent motion correlation may be explained by the visual hierarchy, with lower visual areas (V1, PM, and LM) showing strong motion coherence correlation, but higher areas (AM, RL) showing weaker motion correlation. Note that the negative correlation between the preferred elevation and motion response could not be explained by a decreasing anterior-to-posterior anatomical gradient, as several HVAs (e.g., area PM) have vertical retinotopic gradients that not co-oriented to the anatomical gradient. Furthermore, the negative correlations tended to be strongest in regions in which the pixels subtended a larger range of elevation (Supplementary Fig. 9), as measured by the elevation covered by 95% of the pixels (*E*_95%_), such as V1 (*E*_95%_ = 58.42°) and PM (*E*_95%_ = 32.16°), compared with areas AM (*E*_95%_ = 25.25°) and AL (*E*_95%_ = 20.85°). There was no relationship between azimuth correlations and azimuth coverage (A_95%_; Supplementary Fig. 9C). Although the retinotopic coverage of the HVAs is similar to that reported with intrinsic signal imaging in previous work^40^, it is possible that the population averaging and low-pass filtering inherent to wide-field population imaging approaches may underestimate the retinotopic extent of the individual neurons within each region.

### Coherent motion correlations of single neurons

**Fig. 6:**
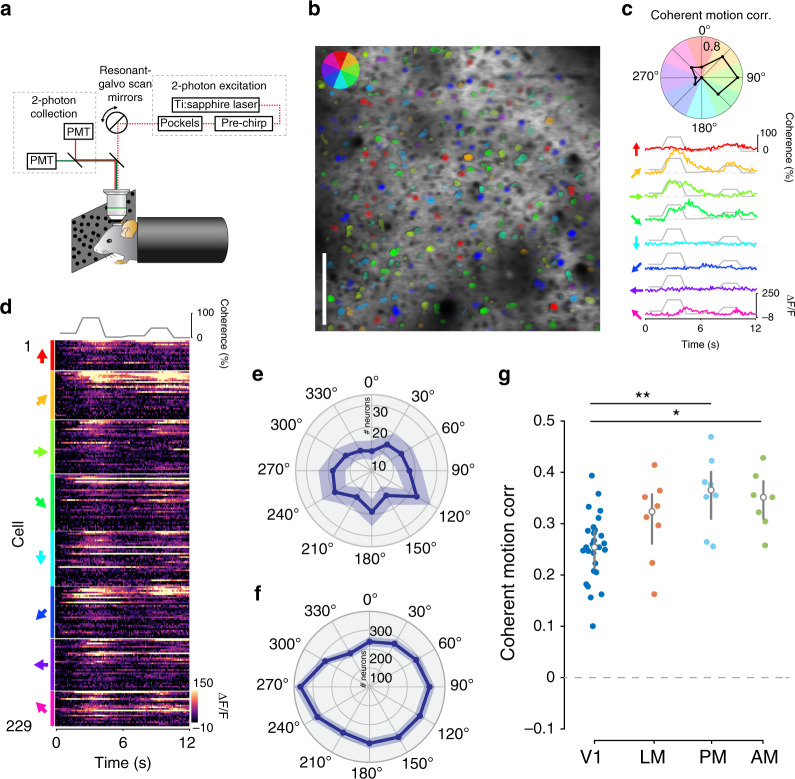
Coherent motion responses of single cells. **a** Schematic of the two-photon microscopy experimental setup. **b** Example two-photon imaging field from V1. Each neuron is color-coded by its preferred direction; the opacity of the color corresponds to its mean activity level. Scale bar is 100 µm. **c** Top: polar plot of coherent motion correlation for each motion direction. Bottom: responses of a single example neuron to each of the eight motion directions, highlighting the direction selectivity of an individual neuron. The coherence trace for this session is overlaid on each neural response. **d** Responses of each cell in the example session, grouped by preferred direction (left, arrows) and ordered by decreasing coherent motion correlation. **e** Polar plot of averaged directional tuning to coherent motion across all cells in the example session. **f** Histogram of 30° binned preferred direction of all neurons across all sessions. Note the significant non-uniformity across all sessions (*n* = 3461 cells from 13 mice, *p* = 1.73 × 10^−6^, Hodges–Ajne test for non-uniformity). **g** Mean coherent motion correlation across HVAs. Each point represents the mean coherent motion correlation of all the cells in that session. Although all areas exhibit significant coherent motion correlations (V1: *p* = 1.6 × 10^−17^; LM: *p* = 1.3 × 10^−5^; PM: *p* = 3.6 × 10^−6^; AM: *p* = 2.2 × 10^−6^), areas AM and PM have significantly higher coherent motion correlations than area V1 (PM: *p* = 1.3 × 10^−2^; AM: *p* = 1.7 × 10^−3^; two-tailed unpaired two-sample *t* test). *p < 0.05; **p < 0.01, two-sample *t* test.

Although wide-field imaging revealed differences in coherent motion correlations both across and within visual regions, we wanted to confirm that individual neurons exhibited the same response distributions. To this end, we used two-photon calcium imaging to measure the activity of populations of Layer 2/3 excitatory neurons in regions V1 and a subset of HVAs (LM, PM, and AM) that exhibited varying levels of coherent motion correlation (Fig. 6a, b). To determine whether individual neurons exhibit coherent motion correlations, we displayed RDKs in eight directions at a range of coherence values (0–100%), then measured the correlation coefficient between the average calcium response and the coherence trace for each direction (Fig. 6c; Supplemental Video S2). We found that individual neurons showed significant coherent motion selectivity in V1 (74% of 11,018 cells, *n* = 27 sessions over 15 mice) and all imaged HVAs, including LM (83% of 2166 cells, *n* = 8 sessions over 8 mice), PM (84% of 1781 cells, *n* = 8 sessions over 8 mice), and AM (82% of 1178 cells, *n* = 7 sessions over 7 mice). As expected, individual neurons exhibited selectivity for particular coherent motion directions^3^ (Fig. 6b, c). As such, across all visual areas, population responses to direction were highly mixed, though there was a slight bias toward the horizontal directions (nasal and temporal; Fig. 6d, e) that was consistent across imaging fields and mice (Fig. 6f; *p* = 1.76 × 10^−6^, *n* = 3461 cells, Hodges–Ajne test for circular non-uniformity). Although the mean coherent motion correlation for the preferred direction was significant for all visual areas imaged (Fig. 6g; V1: 0.26 ± 0.02, *t*_26_ > 20, *p* = 1.6 × 10^−17^; LM: 0.31 ± 0.01, *t*_7_ = 9.7, *p* = 1.3 × 10^−5^; PM: 0.37 ± 0.01, *t*_7_ = 11.8, *p* = 3.6 × 10^−6^; AM: 0.36 ± 0.01, *t*_6_ = 15.6, *p* = 2.2 × 10^−6^; Bonferroni-corrected threshold, *p* < 0.006, mean ± s.e.m., single-sample *t* test), it was significantly higher in areas PM (*t*_33_ = 2.3, *p* = 1.3 × 10^−2^, Hedges’ *g* = 1.2, unpaired two-sample *t* test) and AM (*t*_32_ = 3.2, *p* = 1.7 × 10^−3^, Hedges’ *g* = 1.0, unpaired two-sample *t* test) as compared with V1, similar to results from wide-field imaging (Fig. 3).

### Single neuron asymmetry in coherent motion correlation

**Fig. 7:**
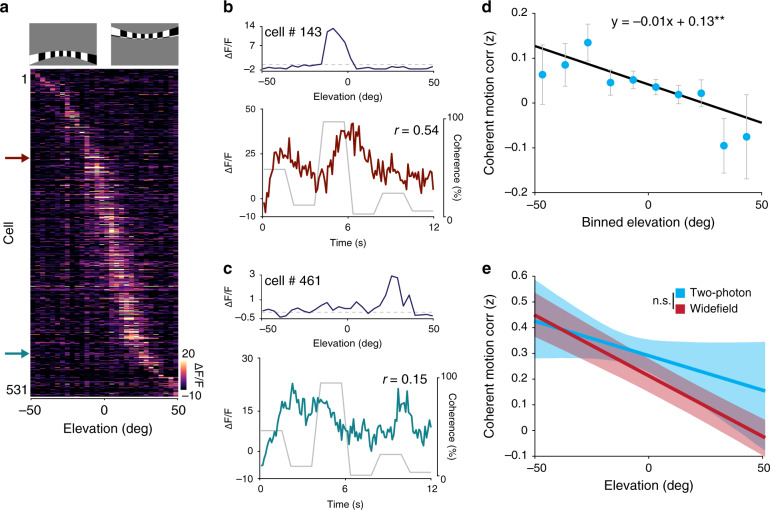
Retinotopic asymmetry of single-cell coherent motion correlations in V1. **a** Top: examples of a high elevation and low elevation spherically corrected retinotopic bar stimulus. Bottom: averaged responses of each cell in the field to the elevation retinotopic bar stimulus, ordered by elevation preference. **b**, **c** Top: the preferred elevation location of the specified cell. Bottom: the calcium response of the cell during the RDK stimulus, with the coherence trace overlaid. **d** Plot of elevation preference versus *z*-scored coherent motion correlation with neurons averaged within 10° elevation bins (*n* = 2680 cells from 13 mice). Error bars are mean ± s.e.m. **e** Plot of mean elevation preference versus coherent motion correlation averaged across experiments for wide-field (red) and two-photon experiments (blue). The confidence band represents the bootstrapped 95% confidence intervals of the slope and intercept (wide field vs. two-photon: *p* = 0.51, two-tailed unpaired two-sample *t* test). **p < 0.01.

Finally, we wanted to confirm that the lower field bias of coherent motion processing evident in the wide-field imaging experiments is also present at the level of individual neurons. To test this, we first measured the preferred azimuth and elevation of populations of V1 neurons using flashing bars with a flickering checkerboard pattern randomly presented at a range of locations (Fig. 7a; see Methods). We then measured the coherent motion correlation in the preferred direction for each neuron (Fig. 7b, c) and correlated the coherent motion correlation with the preferred azimuth and elevation. Similar to the wide-field imaging results (Fig. 4), neurons preferring lower elevation exhibited greater coherent motion correlations than neurons preferring higher elevation (Fig. 7d, e), although the individual neurons exhibited greater variance (Fig. 7d) and the linear fits had a shallower slope than with wide-field imaging (two-photon: −0.63% deg^−1^, wide field: −1.04% deg^−1^). No correlation was present between coherent motion correlation and azimuth (Supplementary Fig. 10). Taken together, the individual neurons largely reflect both the areal differences and retinotopic asymmetry observed wide-field imaging, while also revealing a bias toward horizontal coherent motion that was not evident with mesoscale imaging.

## Discussion

In this paper, we report three key findings: (1) all visual areas measured, including V1, exhibit reliable responses to global coherent motion independent of the spatial content of the retinal image, (2) HVAs respond heterogeneously to coherent visual motion, with stronger responses in putative dorsal stream areas, and (3) neurons which represent the lower visual field having significantly stronger responses to coherent visual motion across visual areas. These findings have implications both for our understanding of murine visual motion processing and for more generalized principles of cortical organization.

In highly visual mammals, researchers have found defined extrastriate regions that are specialized for processing of coherent visual motion, including posteromedial (PMLS) and posterolateral (PLLS) lateral suprasylvian cortex in cats^49,50^, MT and MST cortex in non-human primates^9,12–14^, and V5 in humans^51^. Anatomical^40,43,44^ and functional^23,45^ evidence has suggested that mouse visual cortex might also have dedicated areas specialized for processing of coherent motion, but previous studies had not tested this possibility systematically.

In this paper, we used wide-field calcium imaging to systematically investigate the activity of seven visual areas (V1, LM, AL, PM, LI, RL, and AM) in response to coherent motion stimuli. We found that all visual regions exhibited reliable responses to coherent motion, but that areas AM, AL, and PM exhibited stronger correlations to coherent motion than RL, V1, LM, and LI (Fig. 3). These findings held true at the single-cell level, as shown with two-photon calcium imaging of selected populations (Fig. 6). This is mostly consistent with prior categorization of these regions into putative dorsal (AL, PM, RL, AM) and ventral (LM, LI) streams^40,43–45^. One exception is area RL, which is generally considered a dorsal stream area, but in our study had moderate responses to coherent visual motion (in between ventral and dorsal stream areas) using RDKs, though not natural movies. A recent paper suggests that region RL might be preferentially involved in processing binocular disparity, with inferior responsive neurons particularly attuned to the near field visual stimuli rather than visual motion^52^. Another possibility is that the stimulus parameters used for the RDKs are not well-suited to driving RL neurons, but that coherent motion found in natural movies is more effective.

Although we do not currently know how downstream cortical regions use signals from visual HVAs in local computations, the cortico-cortical connectivity of the motion-responsive regions suggests an intriguing possibility. The HVAs with strong coherent motion correlations (PM, AL, and AM) are highly interconnected with regions involved in navigation, including the parietal cortex, retrosplenial cortex, anterior cingulate cortex, and the presubiculum^44^. Indeed, area AM is often considered a posterior parietal region^43^ though there is not yet widespread agreement on the boundaries between HVAs and parietal cortices^53^. This connectivity profile suggests that neurons in these HVAs may play an important role in visually guided navigation, providing external motion cues to higher cortical regions.

Although we found that mice are similar to cats and primates in having specialized HVAs dedicated to coherent motion processing, we also found a new principal of organization within visual areas that had not been described in previous studies. Specifically, we found that motion processing in mouse visual cortex is asymmetric across elevation, with neurons representing the lower field exhibiting significantly stronger coherent motion correlations both across visual cortex and within defined visual areas (Figs. 5 and 7). We also observed a trend toward a positive correlation in ventral stream areas (LM and LI) between azimuth and coherent motion responsiveness (Fig. 5). However, the positive correlation was not statistically significant, and was not observed in V1 (Fig. 5; Supplementary Fig. 10) or across the visual cortex as a whole (Fig. 4). This is in contrast to the negative correlation between coherent motion correlation and elevation, which was robust across the visual cortex (Fig. 4). The lower field bias is somewhat surprising since it is known that overhead radial motion (such as looming stimuli) can cause strong behavioral reactions such as freezing and escape responses^54^. Indeed, there may even be specialized RGCs for local motion in the superior visual field^29^. This may be due to specialized RGCs responding to different types of motion (radial versus linear). Alternatively, recent studies indicate that behavioral responses to overhead radial motion appear to be principally mediated by a direct circuit from retina to superior colliculus to brain stem nuclei^55,56^. This raises the possibility that motion-responsive RGCs may project to separate thalamic or collicular targets depending on their retinotopy.

It has previously been found that V1 projections to different HVAs exhibit visual response properties consistent with the target area^57^. One way the dorsal stream areas (AL, PM, RL, AM) could inherit both a lower bias in visual field coverage^40^ and strong responses to coherent motion relative to other HVAs would be if they received preferential innervation from anterior V1; with ventral stream areas (LM, LI) receiving preferential input from posterior V1.

An open question is whether there is a functional reason for the lower field bias in coherent motion correlations. One possibility is that coherent motion-responsive units convey external motion cues to downstream regions, which would be consistent with known cortico-cortical connectivity^44^. Since mice have coarse spatial resolution (~10° receptive field size^58^) and their eye level is close to the ground, cues for external motion are likely more prevalent in the lower visual field. That the horizontal bias in preferred direction observed in single neurons (Fig. 6d–f) also reflects a bias in the lower field external motion cues reaching the visual system is consistent with this interpretation.

These results also raise the question of whether the lower field bias of coherent motion correlations might also exist in other visual mammals such as primates. Since primates have higher acuity and their eye level is farther from the ground, it is possible that external motion cues are more distributed across the visual field than for mice, obviating the need for asymmetric processing of motion cues across retinotopic space. However, behavioral evidence of an “lower field advantage” in detecting motion suggests that there may indeed be some lower field bias present even in humans^59,60^, though this has not been confirmed at the cellular level.

It should be noted that although coherent motion correlations were stronger in particular HVAs and in retinotopic regions corresponding to the lower visual field at the population level, there was substantial heterogeneity at the level of individual neurons (Figs. 6 and 7), and that coherent motion-responsive neurons could be found throughout the retinotopic gradient and visual areas.

One of the most surprising findings of the current study is that there is as much variability in coherent motion correlations within a visual area as across visual areas. Several recent studies have found similar mesoscale organization in other response properties in mouse visual cortex. For example, recent studies have found retinotopic asymmetry of color selectivity within area V1^61^ and binocular disparity preference within area RL^52^. Moreover, researchers also found that neurons in higher visual and parietal regions exhibit anatomical gradients of task-modulated responses during navigation in a virtual environment, and that these gradients cross retinotopic borders^62^. Together with our results, this suggests that visual input is not processed uniformly in each area as it progresses through the cortical hierarchy, but rather that there is considerable parallel processing along retinotopic axes within defined areas. Since topographic organization is present in many cortical areas, further research is necessary to determine whether this principle is present in other species and in other sensory, and non-sensory, cortices. These results suggest a general principle of sensory coding, which takes advantage of topographic mapping to selectively route visual information within areas throughout the cortical hierarchy, increasing the bandwidth for ethologically relevant stimuli across cortex.

## Methods

### Animals

For cortex-wide calcium indicator expression, Emx1-Cre (Jax Stock #005628) x ROSA-LNL-tTA (Jax Stock #011008) x TITL-GCaMP6s (Jax Stock #024104) triple transgenic mice (*n* = 25) were bred to express GCaMP6s in cortical excitatory neurons. For wide-field and two-photon imaging experiments, 6–12-week-old mice of both sexes (10 males and 15 females) were implanted with a head plate and cranial window and imaged starting 2 weeks after recovery from surgical procedures and up to 10 months after window implantation. The animals were housed on a 12 h light/dark cycle in cages of up to 5 animals before the implants, and individually after the implants. All animal procedures were approved by the Institutional Animal Care and Use Committee at UC Santa Barbara.

### Surgical procedures

All surgeries were conducted under isoflurane anesthesia (3.5% induction, 1.5–2.5% maintenance). Prior to incision, the scalp was infiltrated with lidocaine (5 mg kg^−1^, subcutaneous) for analgesia and meloxicam (1 mg kg^−1^, subcutaneous) was administered pre-operatively to reduce inflammation. Once anesthetized, the scalp overlying the dorsal skull was sanitized and removed. The periosteum was removed with a scalpel and the skull was abraded with a drill burr to improve adhesion of dental acrylic. A 4 mm craniotomy was made over the visual cortex (centered at 4.0 mm posterior, 2.5 mm lateral to Bregma), leaving the dura intact. A cranial window was implanted over the craniotomy and sealed first with silicon elastomer (Kwik-Sil, World Precision Instruments) then with dental acrylic (C&B-Metabond, Parkell) mixed with black ink to reduce light transmission. The cranial windows were made of two rounded pieces of coverglass (Warner Instruments) bonded with a UV-cured optical adhesive (Norland, NOA61). The bottom coverglass (4 mm) fit tightly inside the craniotomy while the top coverglass (5 mm) was bonded to the skull using dental acrylic. A custom-designed stainless-steel head plate (eMachineShop.com) was then affixed using dental acrylic. After surgery, mice were administered carprofen (5 mg kg^−1^, oral) every 24 h for 3 days to reduce inflammation. The full specifications and designs for head plate and head fixation hardware can be found on our institutional lab website (https://labs.mcdb.ucsb.edu/goard/michael/content/resources).

### Visual stimuli

All visual stimuli were generated with a Windows PC using MATLAB and the Psychophysics toolbox^63^. Stimuli used for wide-field visual stimulation were presented on an LCD monitor (43 × 24 cm, 1600 × 900 pixels, 60 Hz refresh rate) positioned 10 cm from the eye at a 30° angle to the right of the midline, spanning 130° (azimuth) by 100° (elevation) of visual space. The monitor was placed 3 cm above 0° elevation and tilted 20° downward to ensure that the monitor was as equidistant as possible from the mouse’s eye. For two-photon imaging of single-cell responses, visual stimuli were presented on an LCD monitor (17.5 × 13 cm, 800 × 600 pixels, 60 Hz refresh rate) positioned 5 cm from the eye at a 30° angle right of the midline, spanning 120° (azimuth) by 100° (elevation) of visual space. This monitor was also positioned 3 cm above 0° elevation and tilted 20° downward.

Retinotopic mapping stimuli consisted of a drifting bar that was spherically corrected to account for visual field distortions due to the proximity of the monitor to the mouse’s eye^23^. A contrast-reversing checkerboard was presented within the bar to better drive neural activity (0.05 cycles degree^−1^ spatial frequency; 2 Hz temporal frequency). The bar smoothly drifted at 10.8° s^−1^ and had a width of 8° of visual field space for elevation and 9° of visual field space for azimuth. The stimulus was swept in the four cardinal directions: left to right, right to left, bottom to top, and top to bottom, repeated 20–60 times.

Natural movies were a set of 22 home cage movies recorded from mice with head-mounted cameras provided by Froudarakis and Tolias^48^. Each movie had a duration of 10 s and was presented at a frame rate of 30 Hz. For each experiment, three movies were randomly selected from a pool of all movies and presented for 20 repeats in random order.

Random dot kinematograms (RDKs) consisted of black dots presented on a 50% gray screen^64^. Each dot had a diameter of 2° of visual space, and the number of dots was adjusted so that the screen was 20% occupied by dots. Dots moved at a speed of 80° s^−1^ in a randomly assigned direction (0°–315°, in 45° increments), and had a lifetime of 60 frames. A subset of these dots (3, 6, 12, 24, 48, or 96%) had their directions adjusted to the same direction to create coherent motion within the random dot motion. The coherent motion trace, which dictates the amount of coherence in the stimulus, was randomly generated at the beginning of the stimulus presentation and remained constant across repeats. Other stimulus features such as coherence direction and dot position were randomized across trials in order to eliminate reliable responses to all visual features except coherent motion. For wide-field stimulation, a square wave coherence trace consisting of a pseudorandom presentation of all coherence values was created for each experiment, with motion direction changing across repeats. For two-photon stimulation, a short pseudorandom coherence trace was generated, with coherence changing smoothly between target coherence values, and the RDK stimulus was repeated multiple times in different motion directions to allow measurement of directional tuning. A 2 s blank gray screen preceded each trial, and the stimulus was repeated for 10–20 trials. For a subset of experiments, the RDKs were spherically corrected with the same parameters as for the retinotopic mapping stimuli.

To map azimuth and elevation preferences, full screen length bars (width = 20°) of a contrast-reversing checkerboard (spatial frequency of 0.04 cycles degree^−1^; temporal frequency = 5 Hz) were displayed on a 50% gray screen. There were 30 overlapping bar locations for horizontal bars (elevation mapping) and 40 overlapping bar locations for vertical bars (azimuth mapping). The bar appeared at each location in random order for 1 s, with a 2 s gray screen between repeats.

### Wide-field imaging

After >2 weeks of recovery from surgery, GCaMP6s fluorescence was imaged using a custom wide-field epifluorescence microscope. The full specifications and parts list can be found on our institutional lab website (https://labs.mcdb.ucsb.edu/goard/michael/content/resources). In brief, broad spectrum (400–700 nm) LED illumination (Thorlabs, MNWHL4) was band-passed at 469 nm (Thorlabs, MF469-35), and reflected through a dichroic (Thorlabs, MD498) to the microscope objective (Olympus, MVPLAPO 2XC). Green fluorescence from the imaging window passed through the dichroic and a bandpass filter (Thorlabs, MF525-39) to a scientific CMOS (PCO-Tech, pco.edge 4.2). Images were acquired at 400 × 400 pixels with a field of view of 4.0 × 4.0 mm, leading to a pixel size of 0.01 mm pixel^−1^. A custom light blocker affixed to the head plate was used to prevent light from the visual stimulus monitor from entering the imaging path.

### Wide-field post-processing

Images were acquired with pco.edge camera control software and saved into multi-page TIF files. All subsequent image processing was performed in MATLAB (Mathworks). The Δ*F*/*F* for each individual pixel of each image frame was calculated as:

1 $$\begin{array}{*{20}{c}} {\frac{{\Delta F}}{{F_{x,y,n}}} = \frac{{\left( {F_{x,y,n}\, -\, \tilde F_{x,y}} \right)}}{{\tilde F_{x,y}}}} \end{array}$$

where $F_{x,y,n}$ is the fluorescence of pixel (*x, y*) at frame *n*, and $\tilde F_{x,y}$ is defined as the median raw fluorescence value across the entire time series for pixel (*x, y*). Subsequent analyses were performed on whole-frame Δ*F*/*F* matrices.

### Identifying HVAs using wide-field retinotopic mapping

For identification of HVAs, responses to drifting bar stimuli were averaged across each stimulus (horizontal left to right, horizontal right to left, vertical bottom to top, vertical top to bottom)^65^. Next, for each pixel, the phase of the first harmonic of the 1D Fourier transform was calculated to create retinotopic maps (phase maps) for each direction, which were then phase-wrapped to ensure smooth phase transitions between pixels. Last, to remove the delay due to the rise time of the GCaMP6s signal, phase maps of opposite directions (forward vs backward, upward vs downward) were subtracted from one another^65^.

Visual field sign maps were derived from the sine of the angle between the gradients in the azimuth and elevation phase maps. The resulting sign maps underwent a standard post-processing procedure^39,42^: sign maps were first smoothed and thresholded, then each sign patch was dilated to fill in gaps between areas. Next, we applied an iterative splitting and merging process to further refine maps. First, each patch was checked for redundant coverage of visual space, and if significant redundancy (>10% shared visual field coverage) was found, the patch was split to create two separate patches. Conversely, adjacent same-sign patches were merged if they had little redundancy (<10% shared visual field coverage). After processing, borders were drawn around each patch, and resulting patches were compared against published sign maps for both size and sign to label each patch as a visual area. Visual areas V1, LM, AL, PM, LI, RL, and AM were present in all mice (Supplementary Fig. 2).

### Analysis of natural movie and RDK stimuli

For natural movie reliability maps, we calculated the reliability of each pixel according to the following formula:

2 $$\begin{array}{*{20}{c}} {R_{x,y} = \frac{{\mathop {\sum }\nolimits_{t = 0}^T CC\left( {r_{x,y,t},\bar r_{x,y,\left[ {0,T} \right] \ne t}} \right)}}{T}} \end{array}$$

where *R* is reliability for pixel (*x*, *y*), *t* is the trial number from [0,*T*], *CC* is the Pearson correlation coefficient, $r_{x,y,t}$ is the response of pixel (*x*, *y*) on trial *t*, and $\bar r_{x,y,[0,T] \ne t}$ is the average response of pixel (*x*, *y*) on all trials excluding trial *t*.

For calculating coherent motion in natural movies, the pixel-wise motion vectors for each frame were calculated using the MATLAB optical flow toolbox (Mathworks). In brief, the motion vectors are calculated per pixel by analyzing the spatiotemporal changes in brightness via the Horn–Schunck method. For each frame, the component vectors for each pixel were summed, and the magnitude of the summed component vectors was defined as the value of coherent motion for that frame.

In order to determine the uniformity of motion energy across the frame (Fig. S5), we first calculated mean motion magnitude maps by taking the magnitude of each pixel’s motion vector for each frame, then meaning all resulting frames across a single movie. The uniformity of this image was gauged with a uniformity index, based on the ANSI standard for image uniformity. Briefly, the image is first divided into nine equal sections, which tile the image. The average brightness of each area is calculated. The uniformity index is then calculated as follows:

3 $$\begin{array}{*{20}{c}} {{\mathrm{Uniformity}}\,{\mathrm{index}} = 1 - \frac{{B_{{\mathrm{max}}}\, -\, B_{{\mathrm{min}}}}}{{B_{{\mathrm{max}}}\, +\, B_{{\mathrm{min}}}}}} \end{array}$$

where *B* is the brightness of each section. Higher uniformity index values denote a more even image. As the “brightness” of each pixel in the mean magnitude map denotes the strength of motion information, a high uniformity index signifies even motion information across the screen.

For natural movies and RDKs, the coherent motion correlation of each pixel was calculated as the correlation of the mean response of the pixel and the coherent motion of the presented stimulus:

4 $$\begin{array}{*{20}{c}} {M_{x,y} = CC\left( {\bar r_{x,y},m} \right)} \end{array}$$

where *M*_*x,y*_ is the coherent motion response for pixel (*x, y*), *CC* is the Pearson’s correlation coefficient, $\bar r_{x,y}$ is the average adjusted Δ*F*/*F* response for pixel (*x, y*), and *m* is the coherence value of the stimulus.

To combine reliability and coherent motion correlation maps across mice, individual maps were warped to align them to the Allen Brain Institute Common Coordinate Framework (https://scalablebrainatlas.incf.org/mouse/ABA_v3). Warping was performed on individual sign maps using custom code to ensure precise alignment of boundaries (available at https://labs.mcdb.ucsb.edu/goard/michael/content/resources). The image transformation parameters were then applied to the reliability or coherent motion correlation maps to warp them to the aligned sign maps. Warped maps were used for visualization, but all statistical analyses were performed on individual maps, as described below.

Regions of interest for seven visual areas (V1, LM, AL, PM, LI, RL, and AM) were individually defined for each mouse using the mouse-specific sign map as described above^40^. Pixels within each defined area were averaged to compare areal responses across all imaged mice.

The retinotopic dependence of coherent motion correlations was calculated as the correlation between the retinotopic preference (elevation or azimuth) and the coherent motion correlation for all pixels within each area. Density plots were created using scatplot (MATLAB Central File Exchange). Briefly, a subsampled scatter plot was first created, then turned into a Voronoi diagram. The density of points within each Voronoi cell centered on the subsampled scatter plot was used to determine the density of the distribution in that portion of the scatter plot.

### Two-photon imaging

After >2 weeks recovery from surgery, GCaMP6s fluorescence was imaged using a Prairie Investigator two-photon microscopy system with a resonant galvo-scanning module (Bruker). Prior to two-photon imaging, epifluorescence imaging was used to identify the visual area being imaged by aligning to areal maps measured with wide-field imaging.

For fluorescence excitation, we used a Ti:Sapphire laser (Mai-Tai eHP, Newport) with dispersion compensation (Deep See, Newport) tuned to *λ* = 920 nm. For collection, we used GaAsP photomultiplier tubes (Hamamatsu). To achieve a wide field of view, we used a 16×/0.8 NA microscope objective (Nikon) at 1× (850 × 850 μm) or 2× (425 × 425 μm) magnification. Laser power ranged from 40 to 75 mW at the sample depending on GCaMP6s expression levels. Photobleaching was minimal (<1% min^−1^) for all laser powers used. A custom stainless-steel light blocker (eMachineShop.com) was mounted to the head plate and interlocked with a tube around the objective to prevent light from the visual stimulus monitor from reaching the PMTs. During imaging experiments, the polypropylene tube supporting the mouse was suspended from the behavior platform with high tension springs (Small Parts) to reduce movement artifacts.

### Two-photon post-processing

Images were acquired using PrairieView acquisition software and converted into TIF files. All subsequent analyses were performed in MATLAB (Mathworks) using custom code (https://labs.mcdb.ucsb.edu/goard/michael/content/resources). First, images were corrected for X–Y movement by registration to a reference image (the pixel-wise mean of all frames) using 2-dimensional cross correlation.

To identify responsive neural somata, a pixel-wise activity map was calculated using a modified kurtosis measure. Neuron cell bodies were identified using local adaptive threshold and iterative segmentation. Automatically defined ROIs were then manually checked for proper segmentation in a graphical user interface (allowing comparison to raw fluorescence and activity map images). To ensure that the response of individual neurons was not due to local neuropil contamination of somatic signals, a corrected fluorescence measure was estimated according to:

5 $$\begin{array}{*{20}{c}} {F_{{\mathrm{corrected}}}\left( n \right) = F_{{\mathrm{soma}}}\left( n \right) - \alpha \times F_{{\mathrm{neuropil}}}\left( n \right)} \end{array}$$

where *F*_neuropil_ was defined as the fluorescence in the region <30 μm from the ROI border (excluding other ROIs) for frame *n* and *α* was chosen from [0 1] to minimize the Pearson’s correlation coefficient between *F*_corrected_ and *F*_neuropil_. The Δ*F*/*F* for each neuron was then calculated as:

6 $$\begin{array}{*{20}{c}} {\frac{{\Delta F}}{F} = \frac{{\left( {F_n - F_0} \right)}}{{F_0}}} \end{array}$$

Where *F*_*n*_ is the corrected fluorescence (*F*_corrected_) for frame *n* and *F*_0_ defined as the mode of the corrected fluorescence density distribution across the entire time series.

### Analysis of two-photon imaging data

To map azimuth and elevation preferences, responses were measured during presentation of horizontal or vertical bars containing an alternating checkerboard stimulus. To identify neurons as visually responsive, a one-way ANOVA was first performed to screen for significantly preferential responses for specific stimulus locations. For neurons passing this criterion, we fit the responses with a 1D Gaussian model to determine the preferred azimuth and elevation. Finally, the receptive field preference of all significantly responding neurons in the imaging field were correlated to their pixel distance from the edge of the screen to ensure that the imaged neurons had receptive fields on the screen, and that the retinotopic preference correlated with the anatomical axis of azimuth/elevation (to ensure that the imaging field did not cross areal boundaries). Sessions that failed to exhibit a correlation between receptive field preference and pixel distance along the retinotopic axis were not used for further analysis. The cutoff for correlation was calculated for each recorded field by shuffling the cell locations 5000 times and calculating the correlation between receptive field preference and shuffled pixel distance to probe the underlying distribution. The 99th percentile of the shuffled distribution was then chosen as the threshold correlation, and sessions whose calculated correlations were below this value were discarded

For analysis of single neurons to RDKs, we analyzed responses to different motion directions separately. We first only used the motion direction that produced the highest Pearson correlation coefficient between the neural response and the coherent motion signal, as responses to null directions were generally weak. However, to calculate the net preferred direction of the neuron, we treated the coherent motion responses to each direction as a vector and calculated the vector sum of all vectors. The orientation of the resultant vector was defined as the preferred direction of that neuron.

For measuring coherent motion correlations by area, we imaged fields of neurons at 2× magnification (425 μm × 425 μm) in identified visual areas, as identified in wide-field visual field sign maps. We then compared distributions of single-cell coherent motion correlation across areas. For measuring the relationship of coherent motion correlations to retinotopy in single neurons, we calculated the correlation coefficient between the coherent motion correlation and the preferred elevation.

### Eye tracking and center of gaze analyses

To confirm the center of the gaze of the mouse relative to the stimulus monitor, we performed eye tracking experiments on three mice. These mice were head-fixed identically to imaging experiments, but an IR camera (Thorlabs DCC1645C with IR filter removed; Computar T10Z0513CS 5–50 mm f/1.3 lens) was placed such that the image sensor was located exactly at the center of the stimulus monitor. Video was acquired at 10 fps and images were analyzed offline in MATLAB (Mathworks).

First, the pupil was identified for each frame using an automated procedure. In brief, raw images were binarized based on pixel brightness, and the resulting images were morphologically cleaned by removing isolated pixels. For the initial frame, the pupil was manually chosen. For subsequent frames, the pupil was chosen from potential low intensity regions based on a linear combination of size, location, and eccentricity of the pupil in the previous frame. We next calculated the vector that passed through the center of the mouse’s eyeball and the center of the pupil for each frame^66^. Extrapolating this vector to the stimulus monitor distance provided a measurement of the center of gaze of the mouse on the stimulus monitor at each frame.

### Statistical information

To test statistical significance of single groups, single-sample Student’s *t* tests were performed. Because most of the groups were uneven in sample size, unpaired two-sample *t* tests were used exclusively. To calculate effect size where applicable, Hedges’ *g* was calculated due to uneven group sizes. All *t* tests were performed as two-tailed *t* tests. Where applicable, a Bonferroni correction was applied to adjust the *p*-value significance threshold.

### Reporting summary

Further information on research design is available in the Nature Research Reporting Summary linked to this article.

## Supplementary information

Supplementary Information

Reporting Summary

Description of Additional Supplementary Files

Supplementary Movie 1

Supplementary Movie 2

## Data Availability

Most of the hardware designs can be found on our institutional lab website (https://labs.mcdb.ucsb.edu/goard/michael/content/resources). All source data for Figs. 2-d, e, 3c, d, 4d–f, 5a, i, j, 6g, 7d and Supplementary Figs. 1E, 3C, 4A, B, 5C, 6D–F, 7D–F, 8D, E, 9B, C, 10A are included in the Source data file. Raw data for Figs. 2–5 are available on FigShare (10.35092/yhjc.c.5018363). All other raw data are available upon request. Source data are provided with this paper.

## Source data

Source Data

## Code availability

All of the source code is available on the following GitHub page: https://github.com/kevinksit/CoherentMotionProject.  Source data are provided with this paper.

## References

[1] Newsome WT, Britten KH, Salzman CD, Movshon JA (1990). Neuronal mechanisms of motion perception. Cold Spring Harb. Symp. Quant. Biol..

[2] Rasmussen R, Yonehara K (2017). Circuit mechanisms governing local vs. global motion processing in mouse visual cortex. Front. Neural Circuits.

[3] Marques T (2018). A role for mouse primary visual cortex in motion perception. Curr. Biol..

[4] Dyballa L, Hoseini MS, Dadarlat MC, Zucker SW, Stryker MP (2018). Flow stimuli reveal ecologically appropriate responses in mouse visual cortex. Proc. Natl Acad. Sci. USA.

[5] Youngstrom IA, Strowbridge BW (2012). Visual landmarks facilitate rodent spatial navigation in virtual reality environments. Learn. Mem..

[6] Huberman AD, Niell CM (2011). What can mice tell us about how vision works?. Trends Neurosci..

[7] Glickfeld LL, Olsen SR (2017). Higher-order areas of the mouse visual cortex. Annu. Rev. Vis. Sci..

[8] Seabrook TA, Burbridge TJ, Crair MC, Huberman AD (2017). Architecture, function, and assembly of the mouse visual system. Annu. Rev. Neurosci..

[9] Movshon, J. A., Adelson, E. H., Gizzi, M. S. & Newsome, W. T. In *Pattern Recognition Mechanisms* (ed. C. Chagas, R. Gattass, & C. G.) 117–151 (Vatican Press, 1983).

[10] Mishkin M, Ungerleider LG (1982). Contribution of striate inputs to the visuospatial functions of parieto-preoccipital cortex in monkeys. Behav. Brain Res..

[11] Goodale MA, Milner AD (1992). Separate visual pathways for perception and action. Trends Neurosci..

[12] Dubner R, Zeki SM (1971). Response properties and receptive fields of cells in an anatomically defined region of the superior temporal sulcus in the monkey. Brain Res..

[13] Allman JM, Kaas JH (1971). A representation of the visual field in the caudal third of the middle temporal gyrus of the owl monkey (Aotus trivirgatus). Brain Res..

[14] Maunsell JH, Van Essen DC (1983). Functional properties of neurons in middle temporal visual area of the macaque monkey. I. Selectivity for stimulus direction, speed, and orientation. J. Neurophysiol..

[15] Movshon JA, Newsome WT (1996). Visual response properties of striate cortical neurons projecting to area MT in macaque monkeys. J. Neurosci..

[16] Newsome WT, Paré EB (1988). A selective impairment of motion perception following lesions of the middle temporal visual area (MT). J. Neurosci..

[17] Salzman CD, Britten KH, Newsome WT (1990). Cortical microstimulation influences perceptual judgements of motion direction. Nature.

[18] Snowden RJ, Treue S, Andersen RA (1992). The response of neurons in areas V1 and MT of the alert rhesus monkey to moving random dot patterns. Exp. Brain Res..

[19] Simoncelli EP, Heeger DJ (1998). A model of neuronal responses in visual area MT. Vis. Res..

[20] Rust NC, Mante V, Simoncelli EP, Movshon JA (2006). How MT cells analyze the motion of visual patterns. Nat. Neurosci..

[21] Tohmi M, Meguro R, Tsukano H, Hishida R, Shibuki K (2014). The extrageniculate visual pathway generates distinct response properties in the higher visual areas of mice. Curr. Biol..

[22] Wang Q, Burkhalter A (2007). Area map of mouse visual cortex. J. Comp. Neurol..

[23] Marshel JH, Garrett ME, Nauhaus I, Callaway EM (2011). Functional specialization of seven mouse visual cortical areas. Neuron.

[24] Andermann ML, Kerlin AM, Roumis DK, Glickfeld LL, Reid RC (2011). Functional specialization of mouse higher visual cortical areas. Neuron.

[25] Barlow HB, Levick WR (1965). The mechanism of directionally selective units in rabbit’s retina. J. Physiol..

[26] Weng S, Sun W, He S (2005). Identification of ON-OFF direction-selective ganglion cells in the mouse retina. J. Physiol..

[27] Baden T (2016). The functional diversity of retinal ganglion cells in the mouse. Nature.

[28] Dhande OS (2019). Molecular fingerprinting of On–Off direction-selective retinal ganglion cells across species and relevance to primate visual circuits. J. Neurosci..

[29] Zhang Y, Kim I-J, Sanes JR, Meister M (2012). The most numerous ganglion cell type of the mouse retina is a selective feature detector. Proc. Natl Acad. Sci. USA.

[30] El-Danaf RN, Huberman AD (2019). Sub-topographic maps for regionally enhanced analysis of visual space in the mouse retina. J. Comp. Neurol..

[31] Cruz-Martín A (2014). A dedicated circuit links direction-selective retinal ganglion cells to the primary visual cortex. Nature.

[32] Hillier D (2017). Causal evidence for retina-dependent and -independent visual motion computations in mouse cortex. Nat. Neurosci..

[33] Marshel JH, Kaye AP, Nauhaus I, Callaway EM (2012). Anterior-posterior direction opponency in the superficial mouse lateral geniculate nucleus. Neuron.

[34] Piscopo DM, El-Danaf RN, Huberman AD, Niell CM (2013). Diverse visual features encoded in mouse lateral geniculate nucleus. J. Neurosci..

[35] Zhao X, Chen H, Liu X, Cang J (2013). Orientation-selective responses in the mouse lateral geniculate nucleus. J. Neurosci..

[36] Xu X, Ichida J, Shostak Y, Bonds AB, Casagrande VA (2002). Are primate lateral geniculate nucleus (LGN) cells really sensitive to orientation or direction?. Vis. Neurosci..

[37] Muir DR, Roth MM, Helmchen F, Kampa BM (2015). Model-based analysis of pattern motion processing in mouse primary visual cortex. Front. Neural Circuits.

[38] Palagina G, Meyer JF, Smirnakis SM (2017). Complex visual motion representation in mouse area V1. J. Neurosci..

[39] Juavinett AL, Callaway EM (2015). Pattern and component motion responses in mouse visual cortical areas. Curr. Biol..

[40] Garrett ME, Nauhaus I, Marshel JH, Callaway EM (2014). Topography and areal organization of mouse visual cortex. J. Neurosci..

[41] Wekselblatt JB, Flister ED, Piscopo DM, Niell CM (2016). Large-scale imaging of cortical dynamics during sensory perception and behavior. J. Neurophysiol..

[42] Zhuang J (2017). An extended retinotopic map of mouse cortex. Elife.

[43] Wang Q, Gao E, Burkhalter A (2011). Gateways of ventral and dorsal streams in mouse visual cortex. J. Neurosci..

[44] Wang Q, Sporns O, Burkhalter A (2012). Network analysis of corticocortical connections reveals ventral and dorsal processing streams in mouse visual cortex. J. Neurosci..

[45] Smith IT, Townsend LB, Huh R, Zhu H, Smith SL (2017). Stream-dependent development of higher visual cortical areas. Nat. Neurosci..

[46] Chen T-W (2013). Ultrasensitive fluorescent proteins for imaging neuronal activity. Nature.

[47] Madisen L (2015). Transgenic mice for intersectional targeting of neural sensors and effectors with high specificity and performance. Neuron.

[48] Froudarakis E (2014). Population code in mouse V1 facilitates readout of natural scenes through increased sparseness. Nat. Neurosci..

[49] Payne BR (1993). Evidence for visual cortical area homologs in cat and macaque monkey. Cereb. Cortex.

[50] Li B, Li B-W, Chen Y, Wang L-H, Diao Y-C (2000). Response properties of PMLS and PLLS neurons to simulated optic flow patterns. Eur. J. Neurosci..

[51] Zeki S (1991). A direct demonstration of functional specialization in human visual cortex. J. Neurosci..

[52] La Chioma A, Bonhoeffer T, Hübener M (2019). Area-specific mapping of binocular disparity across mouse visual cortex. Curr. Biol..

[53] Lyamzin D, Benucci A (2019). The mouse posterior parietal cortex: anatomy and functions. Neurosci. Res..

[54] Yilmaz M, Meister M (2013). Rapid innate defensive responses of mice to looming visual stimuli. Curr. Biol..

[55] Shang C (2015). A parvalbumin-positive excitatory visual pathway to trigger fear responses in mice. Science.

[56] Evans DA (2018). A synaptic threshold mechanism for computing escape decisions. Nature.

[57] Glickfeld LL, Andermann ML, Bonin V, Reid RC (2013). Cortico-cortical projections in mouse visual cortex are functionally target specific. Nat. Neurosci..

[58] Niell CM, Stryker MP (2008). Highly selective receptive fields in mouse visual cortex. J. Neurosci..

[59] Levine MW, McAnany JJ (2005). The relative capabilities of the upper and lower visual hemifields. Vis. Res..

[60] Zito GA, Cazzoli D, Müri RM, Mosimann UP, Nef T (2016). Behavioral differences in the upper and lower visual hemifields in shape and motion perception. Front. Behav. Neurosci..

[61] Aihara S, Yoshida T, Hashimoto T, Ohki K (2017). Color representation is retinotopically biased but locally intermingled in mouse V1. Front. Neural Circuits.

[62] Minderer M, Brown KD, Harvey CD (2019). The spatial structure of neural encoding in mouse posterior cortex during navigation. Neuron.

[63] Brainard DH (1997). The psychophysics toolbox. Spat. Vis..

[64] Williams DW, Sekuler R (1984). Coherent global motion percepts from stochastic local motions. Vis. Res..

[65] Kalatsky VA, Stryker MP (2003). New paradigm for optical imaging: temporally encoded maps of intrinsic signal. Neuron.

[66] Sakatani, T. & Isa, T. PC-based high-speed video-oculography for measuring rapid eye movements in mice. *Neurosci. Res*. **49**, 123–131 (2004). 10.1016/j.neures.2004.02.002 15099710

