# Supplementary material for: Distributed and retinotopically asymmetric processing of coherent motion in mouse visual cortex
Source: Nat Commun. 2020 Jul 16;11:3565. doi: 10.1038/s41467-020-17283-5 (PMC7366664; doi:10.1038/s41467-020-17283-5)
Supplement: Supplementary file 1 — Supplementary Information [file 41467_2020_17283_MOESM1_ESM.pdf]

SUPPLEMENTARY INFORMATION

Distributed and Retinotopically Asymmetric Processing of Coherent Motion in Mouse Visual Cortex

Kevin K. Sit and Michael J. Goard*

*Correspondence to: michael.goard@lifesci.ucsb.edu

This PDF file includes:

Supplementary Figures 1 to 10

Supplemental Figures

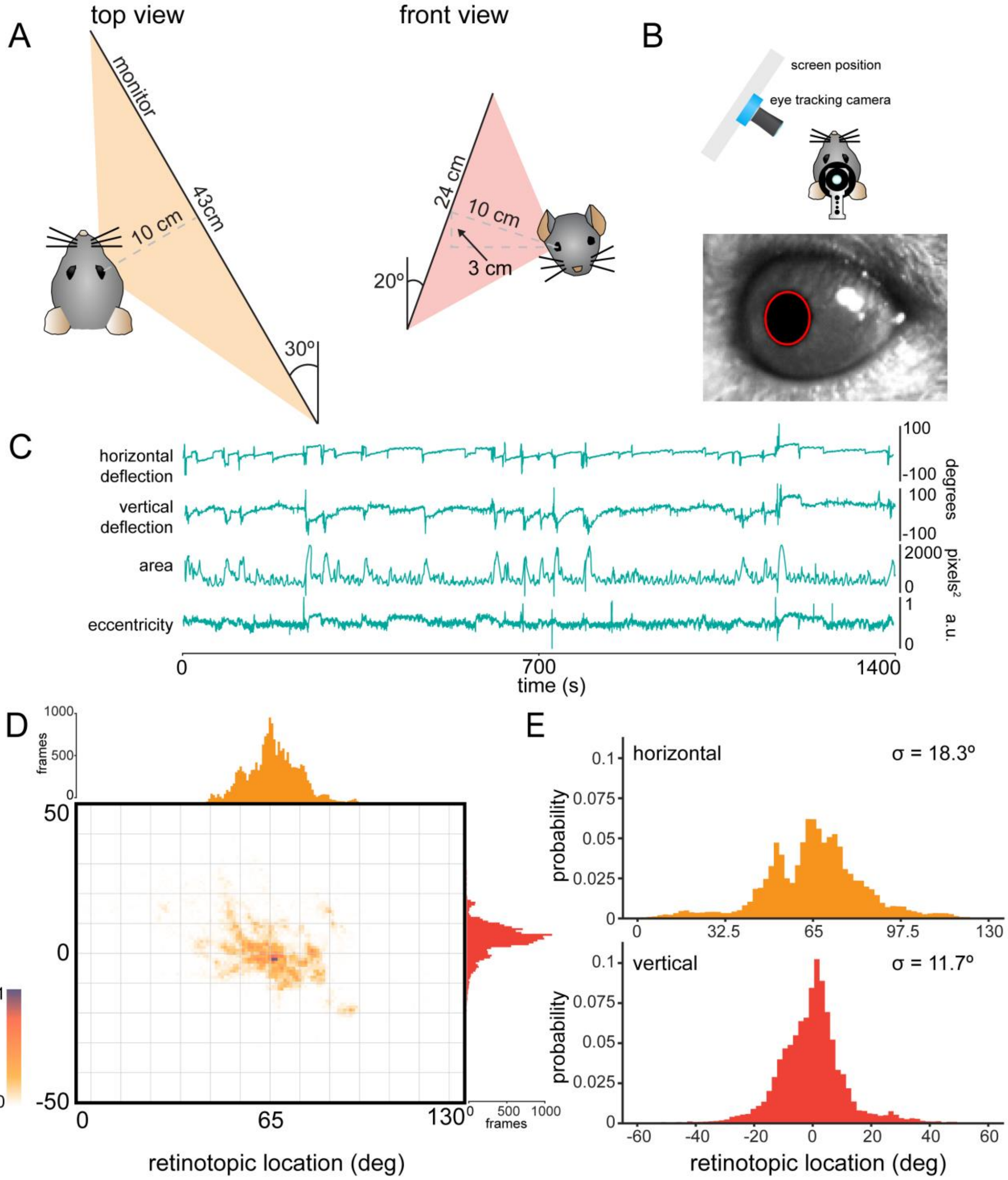

Supplementary Figure 1. Related to Figure 1: Positioning of stimulus monitor and center of gaze tracking

(A) Schematic of monitor placement in front of the mouse from the top and front views (see Methods). **(B)** Schematic of the eye-tracking camera position (top) and an example image (bottom) with the identified pupil circled in red. The sensor of the camera is positioned at the center of the screen location used for visual stimulus presentation. **(C)** Example traces over a 1400 s eye-tracking recording, showing the horizontal deflection, vertical deflection, area, and eccentricity of the detected pupil. **(D)** Density map of center of gaze across the recording for a single mouse. In brief,

the vector representing the center of gaze is extended to the position of the stimulus monitor (see details in Methods). The center of gaze is centered about the center of the screen, with occasional vertical and horizontal deflections. The histograms on the top and right edges of the density map represent the distribution of deflection for azimuth and elevation, respectively. **(E)** Combined probability density histograms across all recorded mice (n = 3). There exists greater spread in the azimuth than elevation direction. Both histograms are centered near the midpoint of the screen.

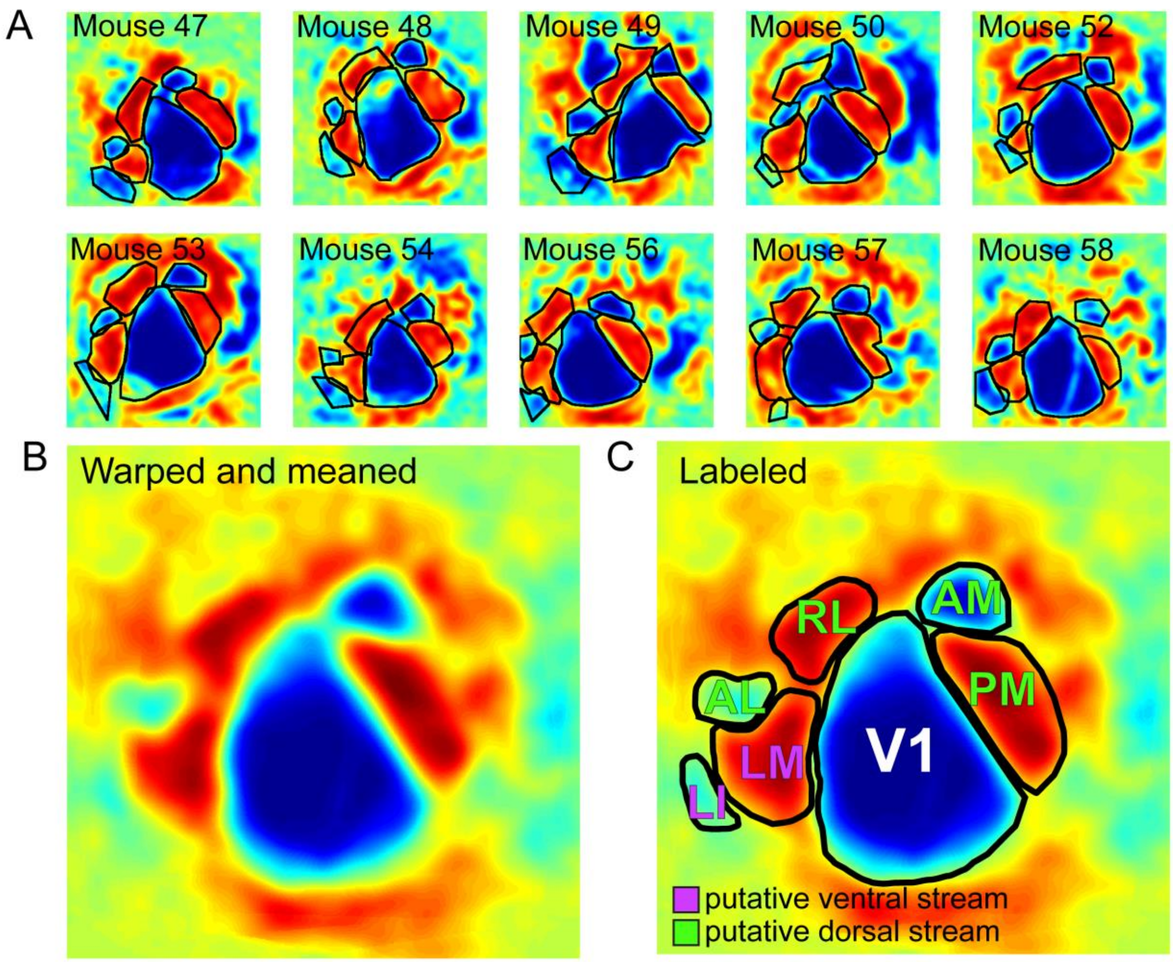

Supplementary Figure 2. Related to Figure 1: Visual field sign maps from all imaged mice

(A) Sign maps for each of ten mice used in both widefield RDK and natural movie experiments. All sign maps contain boundaries which define the eight areas analyzed (V1, LM, PM, AL, LI, RL, AM, and S1). **(B)** Warped and averaged sign maps of all mice. **(C)** Same as in (B), but with areal boundaries and labels included. The putative mouse ventral and dorsal streams are designated in green and magenta, respectively.

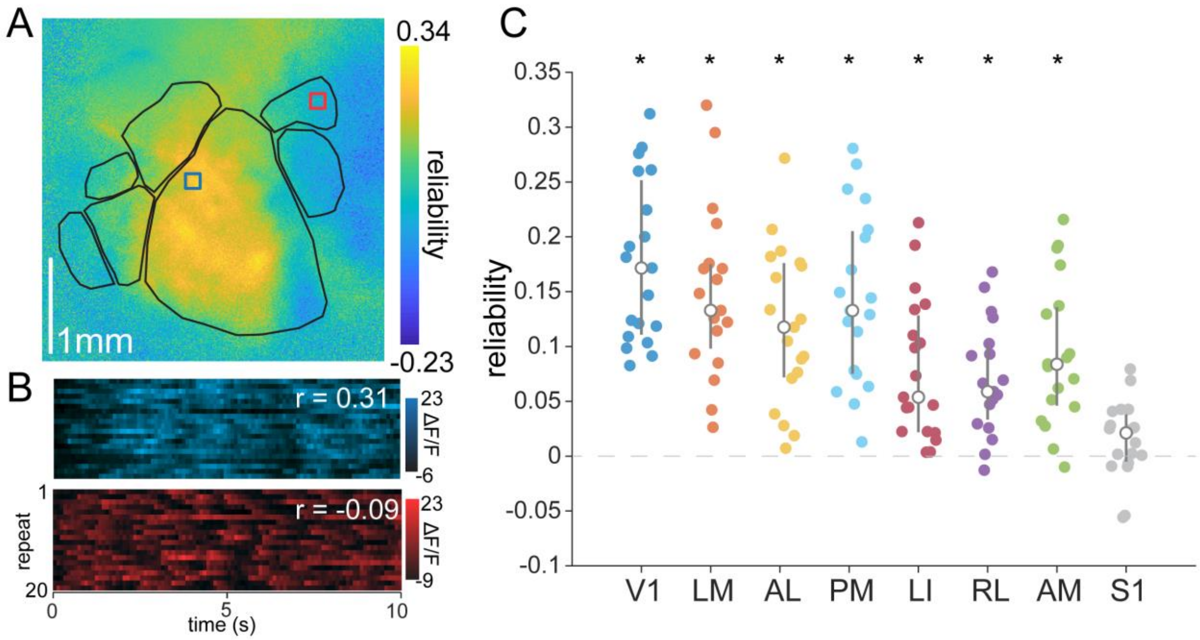

Supplementary Figure 3. Related to Figure 1: Natural movie reliability across mouse visual cortex

(A) Map of reliability to natural movie stimuli. **(B)** Two example pixels from the labeled boxes in (A). The blue pixel shows from area V1 shows high reliability; whereas, the red pixel from area AM shows low reliability. **(C)** V1 and HVAs ordered in approximate ascending hierarchical order, showing a clear decrease in reliability. All areas but area S1 (have significantly reliable visual responses to the natural movies (*:Bonferroni corrected $p < 0.006$, V1: $p = 4.9 \times 10^{-9}$, LM: $p = 1.1 \times 10^{-7}$, PM: $p = 2.5 \times 10^{-7}$, AL: $p = 9.1 \times 10^{-7}$, LI: $p = 4.4 \times 10^{-5}$, RL: $p = 9.3 \times 10^{-6}$, AM: $p = 7.5 \times 10^{-6}$, S1: $p = 0.079$; two-tailed single-sample t-test; $n = 19$ sessions over 7 mice). Error bars are median ± quartiles.

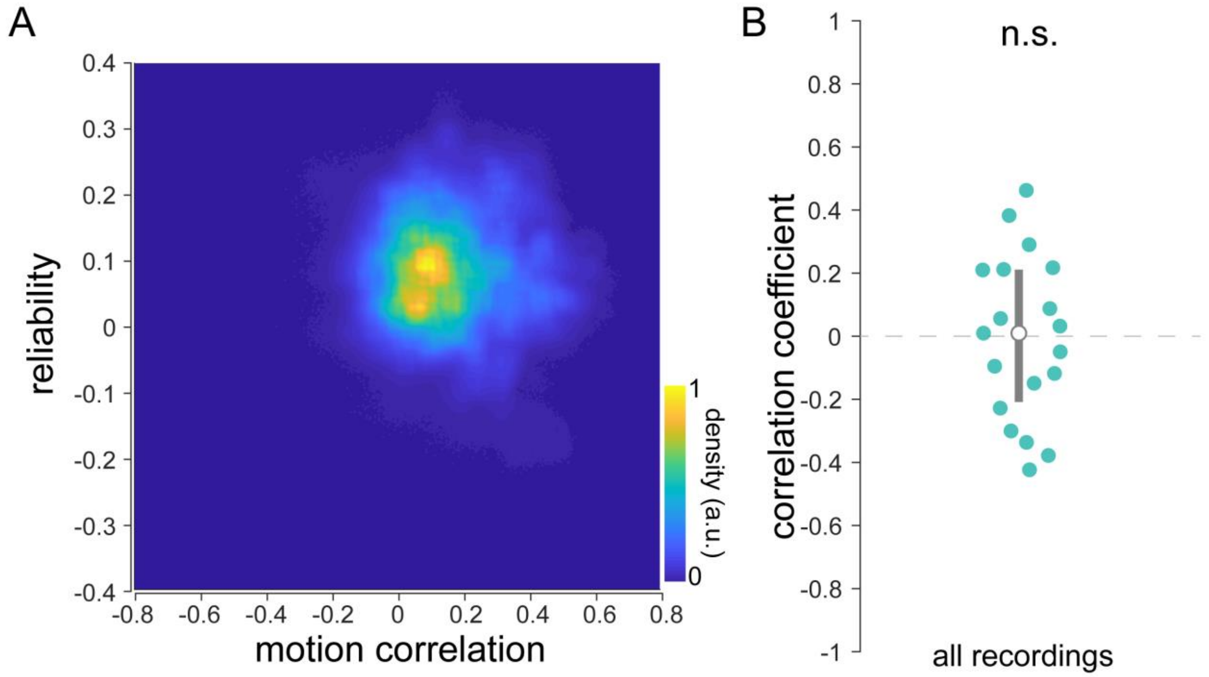

Supplementary Figure 4. Related to Figure 1: Correlation of reliability and motion response of natural movies

(A) Density plot of reliability versus motion correlation for all sessions. **(B)** Correlation between reliability and motion correlation for each session (*n* = 19 sessions over 7 mice). There is no significant correlation between reliability and motion correlation across sessions (*p* = 0.91; two-tailed single-sample t-test). Error bars are median ± quartiles.

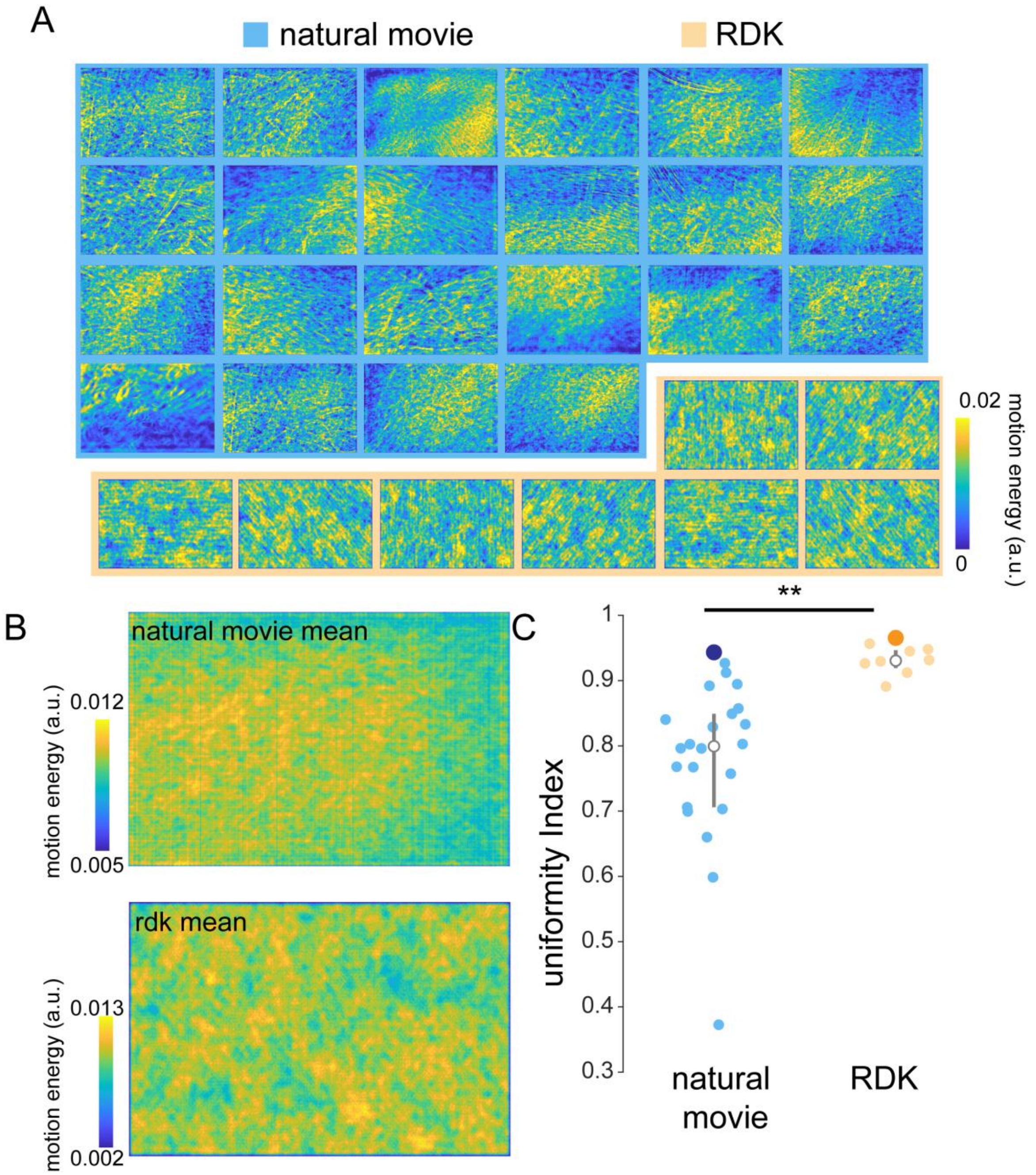

Supplementary Figure 5. Related to Figure 1: Natural movies have uneven motion energy across the frame

(A) Mean motion energy across all frames for each of 22 different natural movies (blue background) and 8 RDKs of all shown directions (orange background). Whereas each natural movie has hotspots of motion energy, RDKs have motion energy present across the entire scene. When displayed across many directions, RDKs have an essentially flat distribution of motion energy across the frame. **(B)** Mean motion energy across all stimuli for natural movies (top) and RDKs (bottom). In both cases, the combination of all stimuli results in a uniform distribution of motion energy across the frame. **(C)** Comparison of uniformity index (see experimental procedures) across the frame for the motion energy of natural movies (n = 22 movies) and RDKs (n = 8 movies). RDKs have much higher motion energy uniformity across the frame (p = 0.004, two-tailed unpaired two sample t-test). For each stimulus, the large dot (dark blue for natural movies, orange for RDKs) is the uniformity index of the single averaged motion energy from (B) (**: $p < 0.01$). Error bars are median ± quartiles.

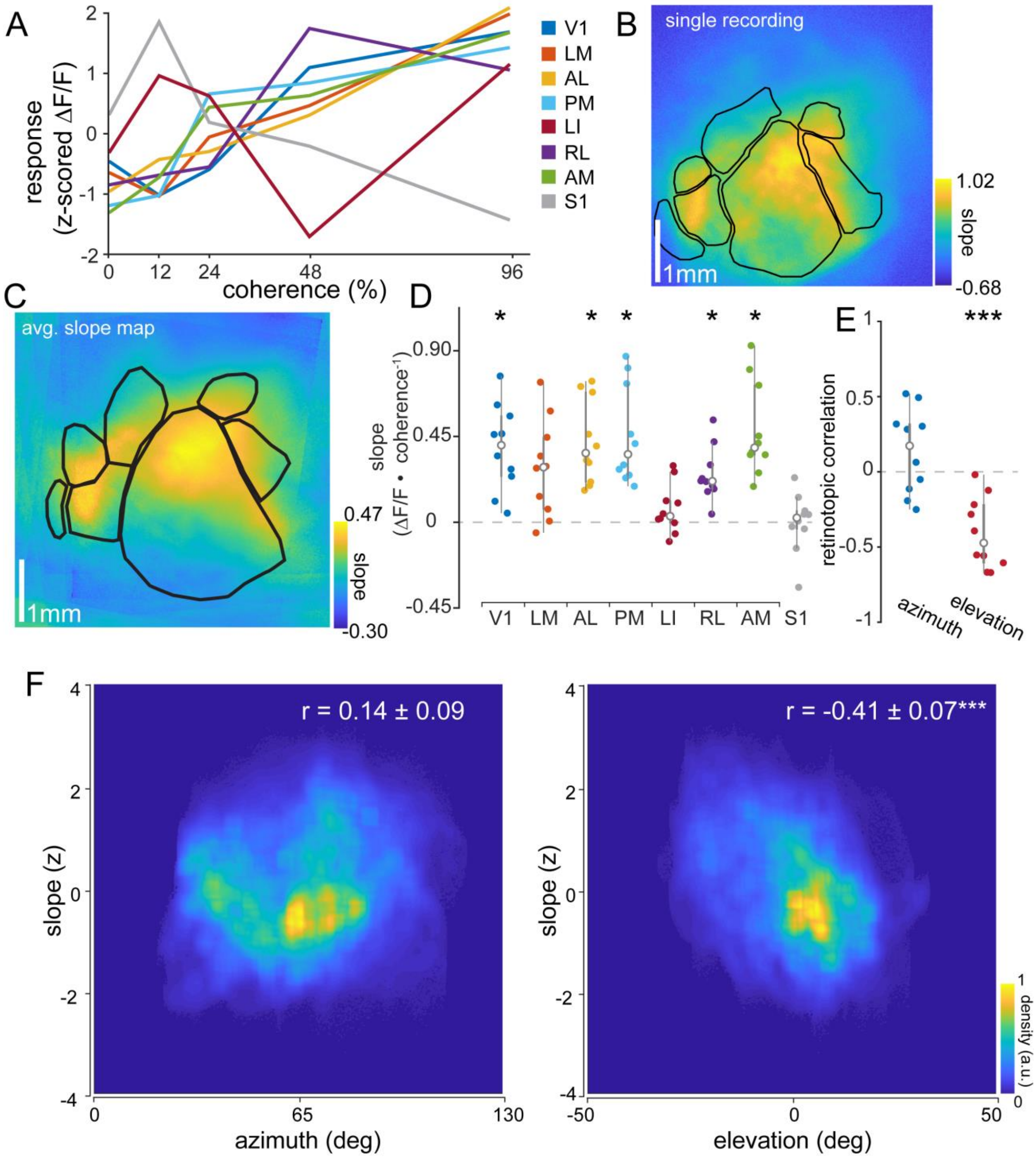

Supplementary Figure 6. Related to Figure 4: Coherent motion response curves measured with mesoscale calcium imaging.

(A) Example of neural responses to coherent motion present in the RDK for each identified cortical area (averaged across all pixels in area). To quantify the coherent motion response, the linear slope was taken for each response curve. **(B)** Map of coherent motion response slopes for a single recording in one single mouse. A higher slope denotes a stronger response to RDKs with higher coherent motion. **(C)** Average slope map across all imaged mice (*n* = 10 sessions over 10 mice). Individual maps are transformed onto a common coordinate system for comparison across mice. **(D)** Across mice (*n* = 10 sessions over 10 mice), dorsal stream areas (areas AL, PM, RL, AM) are significantly responsive to coherent motion, but ventral stream areas (areas LM, LI) and nonvisual areas (area S1)

are not (V1: $p = 4.2 \times 10^{-4}$, LM: $p = 6.4 \times 10^{-3}$, AL: $p = 1.9 \times 10^{-4}$, PM: $p = 3.4 \times 10^{-4}$, LI: $p = 1.2 \times 10^{-1}$, RL: $p = 3.3 \times 10^{-4}$, AM: $p = 1.5 \times 10^{-4}$, S1: $p = 9.6 \times 10^{-1}$, two-tailed single sample t-test) *: Bonferroni corrected $p < 0.006$. Error bars are median ± quartiles. **(E)** Comparison of retinotopic correlation between azimuth ($p = 0.16$, two-tailed single sample t-test) and elevation ($p = 3.9 \times 10^{-4}$, two-tailed single sample t-test) for slope ($n = 10$ sessions over 10 mice). Error bars are median ± quartiles (***: $p < 0.001$). **(F)** Density scatter plot across all mice showing no correlation for azimuth ($p = 0.16$; two-tailed single-sample t-test), but a significant negative correlation for elevation ($p = 3.9 \times 10^{-4}$; two-tailed single-sample t-test).

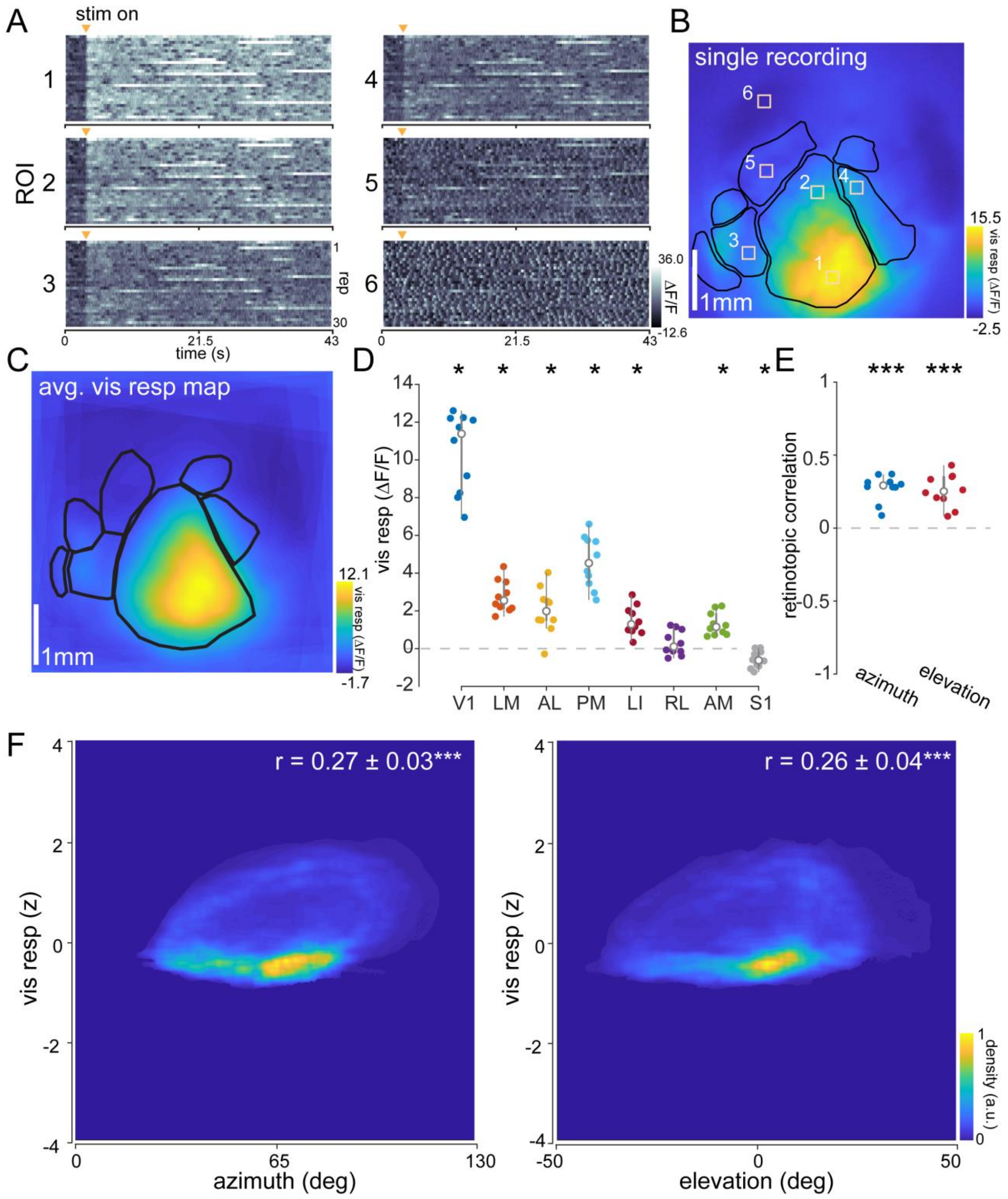

Supplementary Figure 7. Related to Figure 4: Magnitude of visual response to RDK stimulus across posterior cortex

(A) Six example pixels (identified in **B**) showing responses across posterior cortex to entire RDK stimulus set. Visual stimulus prior to stimulus onset (orange arrow) consists of a uniform gray screen. Pixels from visual areas exhibit stronger responses to stimulus onset than pixels outside visual areas. Visual response magnitude (vis resp) is calculated as the difference between the mean on-period activity (post stimulus onset) and the mean off-period

activity (pre stimulus onset). **(B)** Map of visual responsiveness from a single mouse. **(C)** Average visual responsiveness map across all imaged mice (n = 10 sessions over 10 mice). Individual maps are transformed onto a common coordinate system for comparison across mice. **(D)** Across all mice (n = 10 sessions over 10 mice), most visual areas are strongly driven by visual stimuli, whereas S1 shows a mild suppression to visual stimuli (V1: $p = 8.1 \times 10^{-8}$, LM: $p = 2.7 \times 10^{-6}$, AL: $p = 5.4 \times 10^{-4}$, PM: $p = 2.4 \times 10^{-6}$, LI: $p = 2.0 \times 10^{-4}$, RL: $p = 1.8 \times 10^{-1}$, AM: $p = 8.6 \times 10^{-5}$, S1: $p = 3.3 \times 10^{-3}$, two-tailed single sample t-test). Error bars are median ± quartiles (*: Bonferroni corrected $p < 0.006$). **(E)** Comparison of retinotopic correlation between azimuth ($p = 5.2 \times 10^{-6}$, two-tailed single sample t-test) and elevation ($p = 4.7 \times 10^{-5}$, two-tailed single sample t-test) for visual responsiveness (n = 10 sessions over 10 mice). Error bars are median ± quartiles (***: $p < 0.001$). **(F)** Density scatter plot across all mice showing positive correlations for both azimuth ($p = 5.2 \times 10^{-6}$; two-tailed single-sample t-test) and elevation ($p = 4.7 \times 10^{-5}$; two-tailed single sample t-test).

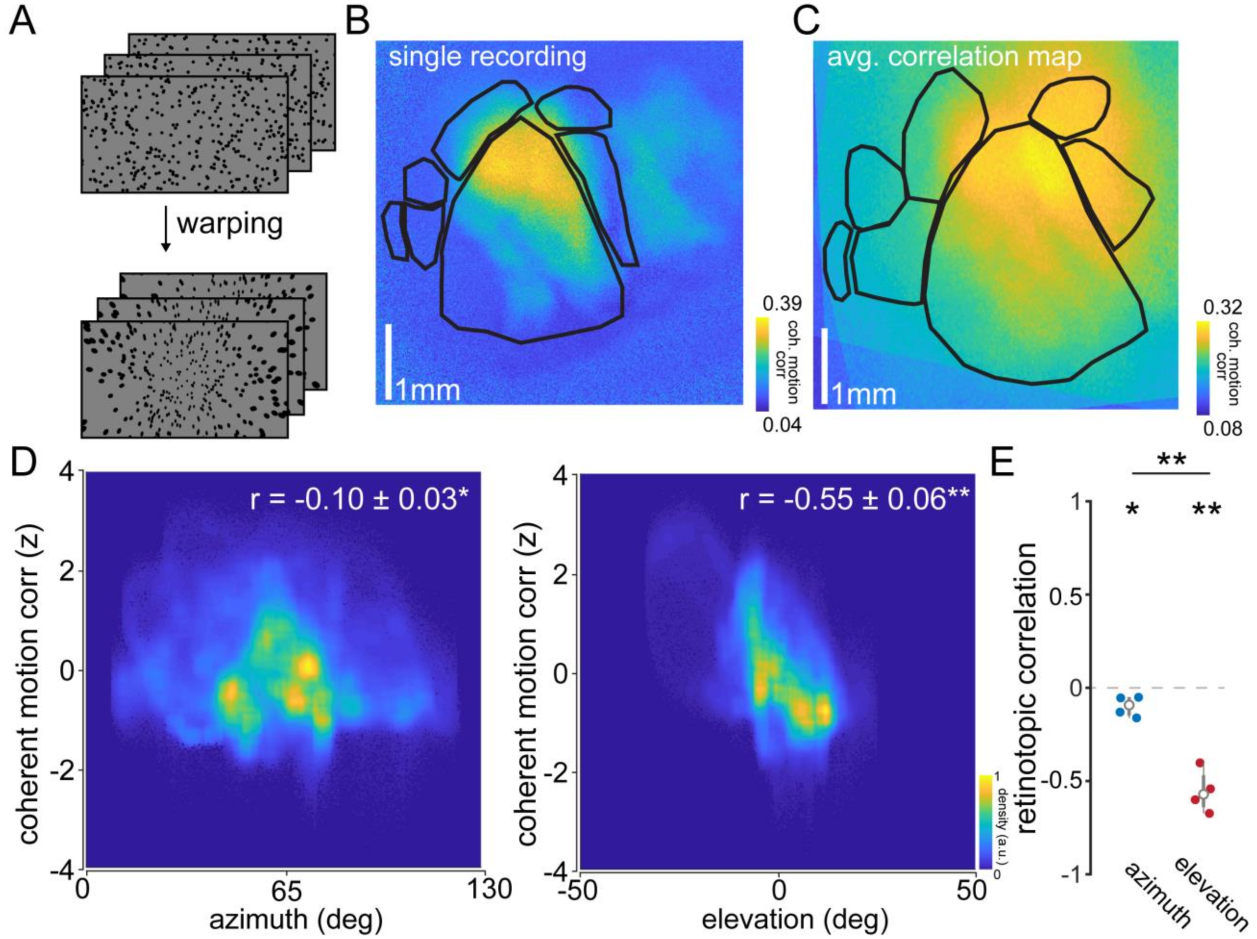

Supplementary Figure 8. Related to Figure 4: Mesoscale calcium responses to coherent motion using spherically corrected RDKs

(A) Schematic of the spherical correction procedure. The same procedure applied to retinotopic mapping stimuli was applied to the RDKs to create warped RDK stimuli. **(B)** Map of coherent motion correlation from a single mouse. **(C)** Average motion correlation map across all imaged mice (*n* = 4 sessions over 4 mice). Individual maps are transformed onto a common coordinate system for comparison across mice. **(D)** Density scatter plot across all mice showing significant correlations for both azimuth (left *r* = -0.10 ± 0.03, *p* = 0.03; two-tailed single-sample t-test) and elevation (right *r* = -0.55 ± 0.06, *p* = 0.002; two-tailed single-sample t-test). However, the retinotopic correlation to elevation is significantly more negative than that for azimuth (*p* = 0.008, two-tailed paired t-test). **(E)** Comparison of retinotopic correlation (*n* = 4 sessions over 4 mice) between azimuth (*p* = 0.03, two-tailed single sample t-test) and elevation (*p* = 0.002, two-tailed single sample t-test). Elevation correlation is significantly more negative than azimuth correlation (*p* = 0.008, two-tailed unpaired two sample t-test). Error bars are median ± quartiles (*: *p* < 0.05, **: *p* < 0.01).

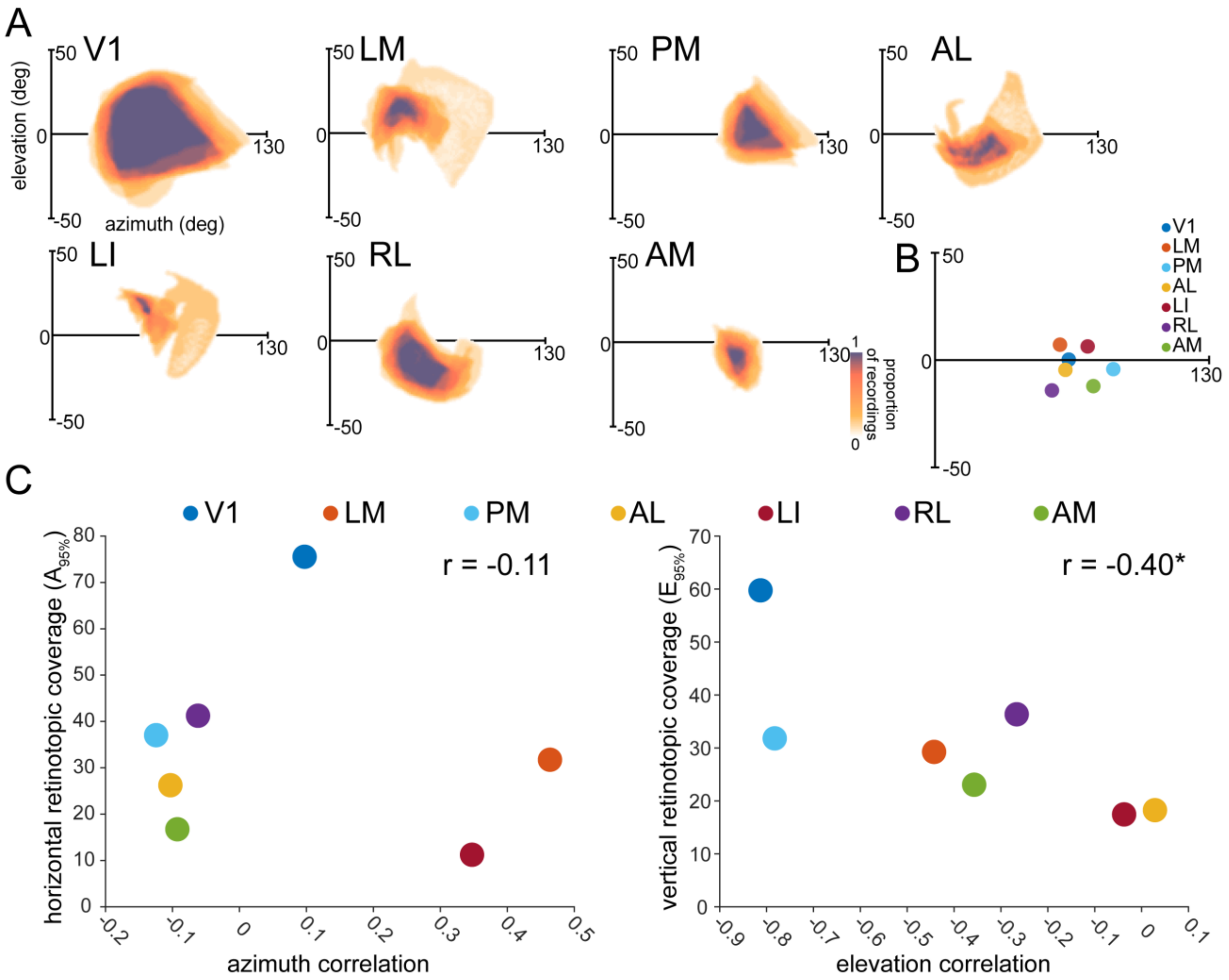

Supplementary Figure 9. Related to Figure 5: Relationship between retinotopic correlation and visual coverage

(A) Visual field coverage of each identified visual area. The center of the axes is the center of V1, denoting (0, 0) in a monocular retinotopic space. **(B)** Centers of each identified area, showing the varying biases of each visual area to different parts of the visual field. **(C)** Comparison between the retinotopic correlation and retinotopic coverage ($A_{95\%}$) in its respective axis for azimuth (left) and altitude (right). Azimuth correlation does not depend on the visual span of each area in the azimuth; whereas, the altitude correlation decreases strongly with decreasing vertical span (azimuth: p = 0.81, altitude: $p = 6.7 \times 10^{-4}$; n = 10 sessions over mice, two-tailed single-sample t-test).

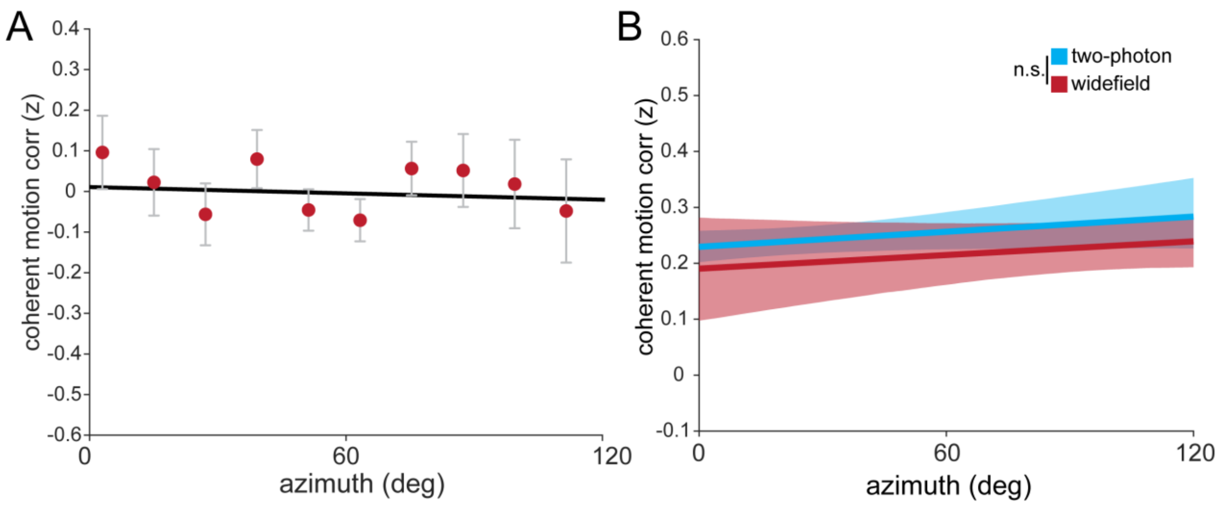

Supplementary Figure 10. Related to Figure 7: Coherent motion responsiveness across azimuth in V1

(A) Plot of azimuth preference versus z-scored coherent motion correlation, averaged in 10° azimuth bins (*n* = 2680 cells from 13 mice). Error bars are mean ± s.e.m. **(B)** Plot of mean azimuth preference versus coherent motion correlation averaged across experiments for widefield (red) and two-photon (blue) experiments, as in Figure 7E. Confidence band represents bootstrapped 95% confidence intervals of slope and intercept. The slopes between widefield and two-photon fit lines are not significantly different ($p = 0.95$, two-tailed single-sample t-test).
