# Supplementary material for: Distributed and retinotopically asymmetric processing of coherent motion in mouse visual cortex
Source: Nat Commun. 2020 Jul 16;11:3565. doi: 10.1038/s41467-020-17283-5 (PMC7366664; doi:10.1038/s41467-020-17283-5)
Supplement: Supplementary file 2 — Reporting Summary [file 41467_2020_17283_MOESM2_ESM.pdf]

nature research

Corresponding author(s): Michael Goard
Last updated by author(s): Jun 12, 2020

Reporting Summary

Nature Research wishes to improve the reproducibility of the work that we publish. This form provides structure for consistency and transparency in reporting. For further information on Nature Research policies, see Authors & Referees and the Editorial Policy Checklist.

Please do not complete any field with "not applicable" or n/a. Refer to the help text for what text to use if an item is not relevant to your study.
For final submission: please carefully check your responses for accuracy; you will not be able to make changes later.

Statistics

For all statistical analyses, confirm that the following items are present in the figure legend, table legend, main text, or Methods section.

n/a	Confirmed	
☐	☒	The exact sample size (*n*) for each experimental group/condition, given as a discrete number and unit of measurement
☐	☒	A statement on whether measurements were taken from distinct samples or whether the same sample was measured repeatedly
☐	☒	The statistical test(s) used AND whether they are one- or two-sided<br>*Only common tests should be described solely by name; describe more complex techniques in the Methods section.*
☒	☐	A description of all covariates tested
☐	☒	A description of any assumptions or corrections, such as tests of normality and adjustment for multiple comparisons
☐	☒	A full description of the statistical parameters including central tendency (e.g. means) or other basic estimates (e.g. regression coefficient) AND variation (e.g. standard deviation) or associated estimates of uncertainty (e.g. confidence intervals)
☐	☒	For null hypothesis testing, the test statistic (e.g. *F*, *t*, *r*) with confidence intervals, effect sizes, degrees of freedom and *P* value noted<br>*Give P values as exact values whenever suitable.*
☒	☐	For Bayesian analysis, information on the choice of priors and Markov chain Monte Carlo settings
☒	☐	For hierarchical and complex designs, identification of the appropriate level for tests and full reporting of outcomes
☐	☒	Estimates of effect sizes (e.g. Cohen's *d*, Pearson's *r*), indicating how they were calculated

Our web collection on statistics for biologists contains articles on many of the points above.

Software and code

Policy information about availability of computer code

Data collection	MATLAB R2018a (Mathworks), Psychophysics Toolbox Version 3 (Open Source), pco.camware V4.10 (pco.), Prairie View V5.4 (Bruker)
Data analysis	MATLAB R2018a (Mathworks), Github: https://github.com/kevinksit/CoherentMotionProject

For manuscripts utilizing custom algorithms or software that are central to the research but not yet described in published literature, software must be made available to editors/reviewers. We strongly encourage code deposition in a community repository (e.g. GitHub). See the Nature Research guidelines for submitting code & software for further information.

Data

Policy information about availability of data

All manuscripts must include a data availability statement. This statement should provide the following information, where applicable:
- Accession codes, unique identifiers, or web links for publicly available datasets
- A list of figures that have associated raw data
- A description of any restrictions on data availability

Most of the hardware designs can be found on our institutional lab website (https://labs.mcdb.ucsb.edu/goard/michael/content/resources). All source data for Figures 2D, E, 3C, D, 4D – F, 5A, I, J, 6G, 7D and Supplementary Figures 1E, 3C, 4A, B, 5C, 6D – F, 7D – F, 8D, E, 9B, C, 10A are included in the Source Data file. Raw data for Figures 2 – 5 are available on FigShare (https://www.doi.org/10.35092/yhjc.c.5018363). All other raw data are available upon request.

Field-specific reporting

Please select the one below that is the best fit for your research. If you are not sure, read the appropriate sections before making your selection.

☒ Life sciences ☐ Behavioural & social sciences ☐ Ecological, evolutionary & environmental sciences

For a reference copy of the document with all sections, see nature.com/documents/nr-reporting-summary-flat.pdf

Life sciences study design

All studies must disclose on these points even when the disclosure is negative.

Sample size	No a priori sample size calculation was performed. Sample sizes were chosen based on previous similar studies (ref. 3, Marques et al 2018).
Data exclusions	For widefield recordings, imaging data were excluded if high quality sign maps were unable to be collected, suggesting a low-quality cortical window. For two-photon recordings, sessions that failed to exhibit a correlation between receptive field preference and pixel distance along the retinotopic axis were not used for further analysis (see Methods). Otherwise, no data were excluded.
Replication	All experiments were replicated in multiple mice (n = 25 mice). Two-photon recordings were repeated within each mouse where indicated up to 4 nonoverlapping recording fields per mouse, but widefield recordings were not. All replicated attempts were successful.
Randomization	Because we are not testing different experimental groups, no randomization was necessary in this study.
Blinding	Data collection and analyses were not performed blind because there were no experimental groups.

Reporting for specific materials, systems and methods

We require information from authors about some types of materials, experimental systems and methods used in many studies. Here, indicate whether each material, system or method listed is relevant to your study. If you are not sure if a list item applies to your research, read the appropriate section before selecting a response.

Materials & experimental systems

n/a	Involved in the study	
☒	☐	Antibodies
☒	☐	Eukaryotic cell lines
☒	☐	Palaeontology
☐	☒	Animals and other organisms
☒	☐	Human research participants
☒	☐	Clinical data

Methods

n/a	Involved in the study	
☒	☐	ChIP-seq
☒	☐	Flow cytometry
☒	☐	MRI-based neuroimaging

Animals and other organisms

Policy information about studies involving animals; ARRIVE guidelines recommended for reporting animal research

Laboratory animals	25 mice (10 males, 15 females) were used. All mice were Emx1-Cre (Jax Stock #005628) x ROSA-LNL-tTA (Jax Stock #011008) x TITL-GCaMP6s (Jax Stock #024104). Mice were singly housed post-surgical procedure in a 12:12 light-dark cycle with the following controlled parameters: temperature (68-76F), humidity(30-70%), ventilation (10-15ACH).
Wild animals	No wild animals were used.
Field-collected samples	No field-collected samples were used.
Ethics oversight	All animal procedures were approved by the Institutional Animal Care and Use Committee at UC Santa Barbara.

Note that full information on the approval of the study protocol must also be provided in the manuscript.

This checklist template is licensed under a Creative Commons Attribution 4.0 International License, which permits use, sharing, adaptation, distribution and reproduction in any medium or format, as long as you give appropriate credit to the original author(s) and the source, provide a link to the Creative Commons license, and indicate if changes were made. The images or other third party material in this article are included in the article's Creative Commons license, unless indicated otherwise in a credit line to the material. If material is not included in the article's Creative Commons license and your intended use is not permitted by statutory regulation or exceeds the permitted use, you will need to obtain permission directly from the copyright holder. To view a copy of this license, visit http://creativecommons.org/licenses/by/4.0/
