# Supplementary material for: Distributed and retinotopically asymmetric processing of coherent motion in mouse visual cortex
Source: Nat Commun. 2020 Jul 16;11:3565. doi: 10.1038/s41467-020-17283-5 (PMC7366664; doi:10.1038/s41467-020-17283-5)
Supplement: Supplementary file 3 — Description of Additional Supplementary Files [file 41467_2020_17283_MOESM3_ESM.pdf]

Description of Additional Supplementary Files

File Name: Supplementary Movie 1
Description: **Widefield responses to retinotopic mapping stimuli** Average widefield $\Delta F/F$ responses during presentation of horizontally or vertically drifting bars used in the retinotopic mapping procedure (scale bar indicates % $\Delta F/F$). The white outlines indicate visual area boundaries based on the sign mapping procedure. Note the multiple waves of propagating activity resulting from discrete retinotopic maps. Inset: retinotopic mapping stimulus shown contralateral to the imaging window.

File Name: Supplementary Movie 2
Description: **Two-photon responses to RDKs** (A) RDK stimulus shown to contralateral eye from two-photon session. (B) Two-photon imaging plane showing multiple cells responsive to the visual stimuli. Inset: Zoomed in view of a single neuron whose trace is highlighted above. (C) Comparison of the coherence signal from the RDK (top) with the neural activity of the highlighted neuron (bottom), showing the tight coupling of neural activity to coherent motion for this neuron.
